# Correction to “Exploring Avenues beyond Revised DSD Functionals: I. Range Separation, with xDSD as a Special Case”

**DOI:** 10.1021/acs.jpca.4c00309

**Published:** 2024-01-30

**Authors:** Golokesh Santra, Minsik Cho, Jan M. L. Martin

In the original reference, we explored the use of range separation as a possible avenue for further improvement on our revised DSD-family functionals. Alongside, we also proposed a family of XYG3^2^ type of double hybrid (i.e., xDSD) as a special case for ω → 0.

Due to a typo found in the “microiteration” input parameter files (i.e., the parameters used in the original electronic structure calculations of GMTKN55^3^), the xDSD- and xDOD-D4 parameters reported in Table 1 of the original reference differ from the actual values by scaling factors. For the actual parameters, see Table 1 here. However, the total WTMAD2 values for each functional and the contributions from the five major subcategories of GMTKN55 remain unchanged (see Table 1 and Table S1 in the Supporting Information.) As a result, updating the parameters does not alter the conclusions drawn from the GMTKN55 data.

**Table 1:** Total WTMAD2 (kcal/mol) and Final Parameters for the xDSD and xOD Functionals with D4 Dispersion Correction

			**Parameters**								
**Functionals**		**WTMAD2**(kcal/mol)	***c***_**X,HF**_	***c***_**C,DFT**_	***c***_**2ab**_	***c***_**2ss**_	***s***_**6**_	***s***_**8**_	***c***_**ATM**_	***a***_**1**_	***a***_**2**_
**xDSD**	**PBEP86**	2.119	0.75	0.3524	0.6894	0.1260	0.4259	[0]	1.0	0.2822	4.7222
	**PBEB95**	2.400	0.74	0.3865	0.6413	0.1139	0.4158	[0]	1.0	0.3533	3.6057
	**BLYP**	2.247	0.77	0.4535	0.6399	0.2566	0.5290	[0]	1.0	0.1362	5.0739
	**SCAN**	2.378	0.69	0.4418	0.6728	0.0328	0.3545	[0]	1.0	0.1915	5.0184
	**PBEPW91**	2.203	0.72	0.4031	0.6724	0.0698	0.5926	[0]	1.0	0.3203	4.1935
	**PBEPBE**	2.238	0.72	0.3976	0.6831	0.0511	0.6108	[0]	1.0	0.3211	4.1784
**xDOD**	**PBEP86**	2.196	0.72	0.3999	0.6743	[0]	0.5395	[0]	1.0	0.2095	5.0154
	**PBEB95**	2.490	0.69	0.4419	0.6075	[0]	0.5158	[0]	1.0	0.3363	3.6147
	**BLYP**	2.543	0.74	0.5174	0.6931	[0]	0.7023	[0]	1.0	0.1080	5.0099
	**SCAN**	2.384	0.69	0.4507	0.6800	[0]	0.3753	[0]	1.0	0.1159	5.4235
	**PBEPW91**	2.219	0.69	0.4462	0.6430	[0]	0.6735	[0]	1.0	0.2679	4.4855
	**PBEPBE**	2.243	0.69	0.4326	0.6453	[0]	0.6875	[0]	1.0	0.2855	4.3563

## Benchmarks External to GMTKN55

As the input parameters for the xDOD and xDOD-D4 functionals are updated, their performance statistics for external benchmarks are also affected. See the original article and references therein for the appropriate references for MOBH35, POLYPYR21, MPCONF196, and CHAL336 test sets. We must note that, apart from the xDSD and xDOD-D4 statistics, everything else is kept unchanged from the original paper.

### a MOBH35

With the corrected parameters, we obtained slightly higher mean absolute errors for the xDSD and xDOD-D4 functionals than previously reported. Among these functionals with D3BJ and D4 dispersion correction, xDODs always outperform the xDSD variants (see Figure S1 in the Supporting Information). xDOD_72_-PBEP86-D4 offers the lowest MAD = 1.2 kcal/mol, close to what was found for the ωDSD_57_-PBEP86-D4(ω=0.22).

Now, excluding the bimolecular reactions also updates the MAD values for the xDOD and xDOD-D4 functionals (see Figure S2 in the Supporting Information). However, the conclusion remains unchanged from the original reference.

### b POLYPYR21

For the xDSD-D4 functionals, updating the parameters increases the MAD and RMSD values compared to what was reported in our original paper, while the corresponding changes for xDOD-D4 are insignificant. With D3BJ and D4 dispersion correction, xDOD functionals perform noticeably better than their xDSD counterparts (see Table S4 in the Supporting Information).

### c MPCONF196

The RMSD values increased marginally upon updating the set of parameters of the xDSD(xDOD)-D4 functionals. As a result, revDSD functionals offer comparable accuracy. For the large subsets, DOD66-SCAN-D4 offers the lowest RMSD (0.64 kcal/mol). Among all the methods tested, revDSD-PBEP86-D4 and revDOD-PBEP86-D4 offer the best accuracy. However, xDSD_75_-PBEP86-D4 and xDOD_72_-PBEP86-D4 are also close competitors (see Figure S5 in the Supporting Information).

### d CHAL336

Similar to the three external sets above, updating the parameters of xDSD75-PBEP86-D4 and xDOD72-PBEP86-D4 increased their mean absolute errors for CHAL336. For all the functionals, a CBS extrapolation significantly improved performance. For the updated MAD numbers, see Table S6 and Figure S3 in the Supporting Information.

## Supporting Information Available

Breakdown of updated total WTMAD2 into top five subsets; contributions from all 55 subsets of GMTKN55; MAD and RMSD for POLYPYR21, MPCONF196, and CHAL336; and figures of MOBH35 error statics for the xDSD and xDOD-D4 functionals (PDF)
