# Supplementary material for: Exploring Avenues beyond Revised DSD Functionals: I. Range Separation, with xDSD as a Special Case
Source: J Phys Chem A. 2021 May 19;125(21):4614–27. doi: 10.1021/acs.jpca.1c01294 (PMC8279641; doi:10.1021/acs.jpca.1c01294)
Supplement: Supplementary file 1 — jp1c01294_si_001.pdf [file jp1c01294_si_001.pdf]

Electronic Supporting Information (ESI)

# Exploring Avenues Beyond Revised DSD Functionals: I. range separation, with xDSD as a special case

*Golokesh Santra,* [†] *Minsik Cho,* [†,§] *and Jan M.L. Martin*[*,†]

[†]Department of Organic Chemistry, Weizmann Institute of Science, 7610001 Reḥovot, Israel.
Email: gershom@weizmann.ac.il

[§]Department of Chemistry, Brown University, Providence, Rhode Island 02912, USA

# SI.1 Details of all 55 subsets of GMTKN55:

Below are the abbreviations used and concise description of all fifty-five subsets of GMTKN55.[1]

**Table S1: Abbreviations used and their descriptions**

| Abbreviation | Description |
|---|---|
| **ACONF**[2] | Relative energies of alkane conformers |
| **ADIM6**[3] | Interaction energies of n-alkane dimers |
| **AHB21**[4] | Interaction energies in anion–neutral dimers |
| **AL2X6**[1] | Dimerisation energies of AlX3 compounds |
| **ALK8**[1] | Dissociation and other reactions of alkaline compounds |
| **ALKBDE10**[5] | Dissociation energies in group-1 and -2 diatomics |
| **AMINO20X4**[6] | Relative energies in amino acid conformers |
| **BH76RC**[7] | 30 reaction energies of the BH76[8–10] set |
| **BH76**[8–10] | Barrier heights of hydrogen transfer, heavy atom transfer, nucleophilic substitution, unimolecular and association reactions |
| **BHDIV10**[1] | Diverse reaction barrier heights |
| **BHPERI**[1,11,12,13] | Barrier heights of pericyclic reactions |
| **BHROT27**[1] | Barrier heights for rotation around single bonds |
| **BSR36**[14,15] | Bond-separation reactions of saturated hydrocarbons |
| **BUT14DIOL**[16] | Relative energies in butane-1,4-diol conformers |
| **C60ISO**[17] | Relative energies between $C_{60}$ isomers |
| **CARBHB12**[1] | Hydrogen-bonded complexes between carbene analogues and H2O, NH3, or HCl |
| **CDIE20**[18] | Double-bond isomerisation energies in cyclic systems |
| **CHB6**[4] | Interaction energies in cation–neutral dimers |
| **DARC**[7,19] | Reaction energies of Diels–Alder reactions |
| **DC13**[20,7,21,22–30] | 13 difficult cases for DFT methods |
| **DIPCS10**[1] | Double-ionisation potentials of closed-shell systems |
| **FH51**[31,32] | Reaction energies in various (in-)organic systems |
| **G21EA**[7,33] | Adiabatic electron affinities |
| **G21IP**[7,33] | Adiabatic ionisation potentials |
| **G2RC**[7,34] | Reaction energies of selected G2/97 systems |
| **HAL59**[35,36] | Binding energies in halogenated dimers (incl. halogen bonds) |
| **HEAVY28**[9] | Noncovalent interaction energies between heavy element hydrides |
| **HEAVYSB11**[1] | Dissociation energies in heavy-element compounds |
| **ICONF**[1] | Relative energies in conformers of inorganic systems |
| **IDISP**[7,37–40] | Intramolecular dispersion interactions |
| **IL16**[4] | Interaction energies in anion–cation dimers |
| **INV24**[41] | Inversion/racemisation barrier heights |
| **ISO34**[37] | Isomerisation energies of small and medium-sized organic molecules |
| **ISOL24**[42] | Isomerisation energies of large organic molecules |
| **MB16-43**[1] | Decomposition energies of artificial molecules |
| **MCONF**[43] | Relative energies in melatonin conformers |
| **NBPRC**[7,39,44] | Oligomerisations and H2 fragmentations of NH3/BH3 systems; H2 activation reactions with PH3/BH3 systems |
| **PA26**[1] | Adiabatic proton affinities (incl. of amino acids) |
| **PArel**[1] | Relative energies in protonated isomers |
| **PCONF21** | Relative energies in tri- and tetrapeptide conformers |
| **PNICO23**[45] | Interaction energies in pnicogen-containing dimers |
| **PX13**[46] | Proton-exchange barriers in H2O, NH3, and HF clusters |
| **RC21**[1] | Fragmentations and rearrangements in radical cations |

| | |
|---|---|
| **RG18**[1] | Interaction energies in rare-gas complexes |
| **RSE43**[47] | Radical-stabilisation energies |
| **S22**[48] | Binding energies of noncovalently bound dimers |
| **S66**[49] | Binding energies of noncovalently bound dimers |
| **SCONF**[7,50] | Relative energies of sugar conformers |
| **SIE4X4**[51] | Self-interaction-error related problems |
| **TAUT15**[1] | Relative energies in tautomers |
| **UPU23**[52] | Relative energies between RNA-backbone conformers |
| **W4-11**[53] | Total atomisation energies |
| **WATER27**[54] | Binding energies in (H2O)n, H+(H2O)n and OH−(H2O)n |
| **WCPT18**[55] | Proton-transfer barriers in uncatalysed and water-catalysed reactions |
| **YBDE18**[56] | Bond-dissociation energies in ylides |

## SI.2. Is inclusion of many body dispersion correction beyond three body ATM term beneficial for revDSD functionals?

In our previous study,[58] for technical reasons we adopted $c_{ATM}=s_6$, where $c_{ATM}$ is the prefactor for the Axilrod-Teller-Muto (ATM)[75,76] 3-body correction term. Here we show, how scaling only three body term separately improve performances further for revDSD functionals (see Table S3). For DSD and DOD-SCAN-D4 this improvement is most prominent, whereas for other cases it is not so outspoken. However, the optimum $s_8$ for all other functionals settled near *zero* and $c_{ATM}$ near *one*. So, if we impose $s_8$=0 and $c_{ATM}$=1 into the *microiteration* cycle, we practically loose no accuracy in terms of WTMAD2 (~0.02 kcal/mol or less).

Our next query is, whether inclusion of the many-body term of dispersion (MBD) beyond ATM is still helpful? From the WTMAD2 values in Table S3 we can conclude that, considering MBD term does more harm than good for revDSD-BLYP-D4, whereas for DSD and DOD-PBEB95-D4 it helps. For other XC combinations, the improvement is marginal.

**Table S2: WTMAD2 values and optimized parameters for different functionals with D4 dispersion correction.**

| Functionals | | WTMAD2(kcal/mol) | | | | Final Parameters | | | | | | | | |
|---|---|---|---|---|---|---|---|---|---|---|---|---|---|---|
| | | revD4 old‡ | revD4 | revD4 $s_8$=0 | revD4 $s_8$=0 $c_{ATM}$=1 | $c_{X,HF}$ | $c_{C,DFT}$ | $c_{2ab}$ | $c_{2ss}$ | $s_6$ | $s_8$ | $c_{ATM}$ ($c_{MBD}$) | $a_1$ | $a_2$ |
| with ATM | DSD-SCAN | 2.577 | 2.458 | 2.474 | 2.477 | 0.66 | 0.4715 | 0.6406 | 0.0155 | 0.4012 | [0] | [1.0] | 0.2548 | 4.5058 |
| | DSD-BLYP | 2.535 | 2.428 | 2.428 | 2.428 | 0.71 | 0.5231 | 0.5578 | 0.1891 | 0.6808 | [0] | [1.0] | 0.1565 | 4.8781 |
| | DSD-PBEPW91 | — | 2.312 | 2.311 | 2.311 | 0.67 | 0.4622 | 0.5861 | 0.0357 | 0.7219 | [0] | [1.0] | 0.3679 | 3.9422 |
| | DSD-PBEB95 | 2.632 | 2.559 | 2.568 | 2.568 | 0.66 | 0.4686 | 0.5197 | 0.0649 | 0.5298 | [0] | [1.0] | 0.3345 | 3.7291 |
| | DSD-PBEPBE | 2.435 | 2.393 | 2.396 | 2.393 | 0.68 | 0.4376 | 0.6108 | 0.0240 | 0.7296 | [0] | [1.0] | 0.5457 | 2.9900 |
| | DSD-PBEP86 | 2.274 | 2.246 | 2.246 | 2.2474 | 0.69 | 0.4224 | 0.5935 | 0.0566 | 0.5917 | [0] | [1.0] | 0.3710 | 4.2014 |
| with MBD | DSD-SCAN | | 2.423 | | | 0.66 | 0.4707 | 0.6462 | 0.0149 | 0.1908 | 0.8382 | 2.7886 | 0.5739 | 3.8328 |
| | DSD-BLYP | | 2.507 | | | 0.71 | 0.5370 | 0.5457 | 0.2032 | 0.4978 | 0.2221 | 0.6780 | 0.2847 | 4.4000 |
| | DSD-PBEPW91 | | 2.301 | | | 0.67 | 0.4706 | 0.5881 | 0.0078 | 0.5912 | 0.4418 | 1.7278 | 0.5476 | 3.2814 |
| | DSD-PBEB95 | | 2.442 | | | 0.66 | 0.4737 | 0.5346 | 0.0343 | 0.3370 | 0.5567 | 2.3138 | 0.6015 | 2.7941 |
| | DSD-PBEPBE | | 2.368 | | | 0.68 | 0.4463 | 0.6122 | 0.0033 | 0.4585 | 0.8390 | 2.1183 | 0.7397 | 2.4901 |
| | DSD-PBEP86 | | 2.276 | | | 0.69 | 0.4252 | 0.5926 | 0.0551 | 0.5343 | 0.0401 | 0.7362 | 0.3604 | 4.2306 |
| with ATM | DOD-SCAN | 2.578 | 2.460 | 2.468 | 2.479 | 0.66 | 0.4780 | 0.6433 | [0] | 0.4088 | [0] | [1.0] | 0.2571 | 4.5012 |
| | *DOD-BLYP* | 2.737 | 2.612 | 2.612 | 2.622 | *0.71* | 0.5548 | 0.6297 | [0] | 0.7661 | [0] | [1.0] | 0.1462 | 4.7790 |
| | DOD-PBEPW91 | — | 2.315 | 2.315 | 2.316 | 0.67 | 0.4669 | 0.5967 | [0] | 0.7436 | [0] | [1.0] | 0.3688 | 3.8960 |
| | DOD-PBEB95 | 2.645 | 2.583 | 2.583 | 2.587 | 0.66 | 0.4799 | 0.5472 | [0] | 0.5548 | [0] | [1.0] | 0.3319 | 3.6735 |
| | DOD-PBEPBE | 2.439 | 2.398 | 2.396 | 2.396 | 0.68 | 0.4449 | 0.6161 | [0] | 0.7438 | [0] | [1.0] | 0.5376 | 3.0254 |
| | DOD-PBEP86 | 2.305 | 2.266 | 2.267 | 2.271 | 0.69 | 0.4301 | 0.6131 | [0] | 0.6158 | [0] | [1.0] | 0.3440 | 4.2427 |
| with MBD | DOD-SCAN | | 2.432 | | | 0.66 | 0.4740 | 0.6477 | [0] | 0.2509 | 0.4330 | 1.7060 | 0.4740 | 3.8328 |
| | DOD-BLYP | | 2.688 | | | 0.71 | 0.5623 | 0.6244 | [0] | 0.5644 | 0.1069 | 0.5561 | 0.1603 | 4.4327 |
| | DOD-PBEPW91 | | 2.298 | | | 0.67 | 0.4691 | 0.5961 | [0] | 0.5114 | 0.6384 | 1.8187 | 0.5945 | 3.1249 |
| | DOD-PBEB95 | | 2.443 | | | 0.66 | 0.4775 | 0.5495 | [0] | 0.3083 | 0.6255 | 2.2835 | 0.6128 | 2.6867 |
| | DOD-PBEPBE | | 2.367 | | | 0.68 | 0.4477 | 0.6142 | [0] | 0.4328 | 0.9006 | 2.1241 | 0.7444 | 2.4918 |
| | DOD-PBEP86 | | 2.296 | | | 0.69 | 0.4324 | 0.6097 | [0] | 0.5226 | 0.0799 | 0.7861 | 0.3833 | 3.9385 |

‡Parameters taken from ref[58]

# SI.3. <u>Diet-GMTKN55 prescreening</u>:

Table S3: WTMAD2 value for different ω and cX,HF(=n) for $\omega DSD_n$ and $\omega DOD_n$-XC-D3BJ [XC=PBEP86, PBEB95, PBEPBE, PBEPW91]

| cX | ω | WTMAD2(kcal/mol) | |
|---|---|---|---|
| | | ωDSD-PBEP86-D3BJ | ωDOD-PBEP86-D3BJ |
| 0.57 | 0.00 | 2.582 | 2.574 |
| | 0.20 | 1.996 | 1.994 |
| | 0.22 | 1.977 | 1.977 |
| | 0.24 | 1.976 | 1.993 |
| | 0.26 | 2.015 | 2.042 |
| 0.60 | 0.00 | 2.438 | 2.425 |
| | 0.15 | 2.004 | 1.991 |
| | 0.18 | 1.940 | 1.937 |
| | 0.20 | 1.922 | 1.917 |
| | 0.22 | 1.924 | 1.929 |
| | 0.25 | 1.959 | 1.997 |
| | 0.30 | 2.080 | 2.166 |
| | 0.35 | 2.196 | 2.355 |
| 0.63 | 0.00 | 2.292 | 2.230 |
| | 0.10 | 2.023 | 1.988 |
| | 0.12 | 1.968 | 1.943 |
| | 0.14 | 1.901 | 1.901 |
| | 0.16 | 1.880 | 1.880 |
| | 0.20 | 1.879 | 1.880 |
| | 0.22 | 1.883 | 1.908 |
| 0.66 | 0.00 | 2.062 | 2.062 |
| | 0.10 | 1.875 | 1.865 |
| | 0.12 | 1.844 | 1.842 |
| | 0.14 | 1.836 | 1.836 |
| | 0.16 | 1.844 | 1.833 |
| | 0.18 | 1.839 | 1.839 |
| | 0.20 | 1.852 | 1.863 |
| | 0.22 | 1.866 | 1.915 |
| | 0.24 | 1.910 | 1.986 |
| 0.69 | 0.00 | 1.973 | 1.936 |
| | 0.10 | 1.804 | 1.799 |
| | 0.12 | 1.801 | 1.795 |
| | 0.14 | 1.800 | 1.795 |
| | 0.16 | 1.800 | 1.806 |
| | 0.18 | 1.818 | 1.834 |
| | 0.20 | 1.841 | 1.893 |
| | 0.22 | 1.877 | 1.961 |
| | 0.30 | 2.092 | 2.252 |
| 0.72 | 0.00 | 1.861 | 1.861 |
| | 0.10 | 1.778 | 1.768 |
| | 0.12 | 1.776 | 1.777 |
| | 0.14 | 1.789 | 1.800 |
| | 0.16 | 1.808 | 1.838 |

Table S3: (Continued)

| cX | w | WTMAD2(kcal/mol) | | | | | |
|---|---|---|---|---|---|---|---|
| | | wDSD-PBEPW91-D3BJ | wDSD-PBEB95-D3BJ | wDSD-PBEPBE-D3BJ | wDOD-PBEPW91-D3BJ | wDOD-PBEB95-D3BJ | wDOD-PBEPBE-D3BJ |
| 0.57 | 0.00 | 2.579 | 3.107 | 2.643 | 2.541 | 3.106 | 2.640 |
| | 0.12 | 2.394 | 3.060 | 2.466 | 2.394 | 3.054 | 2.465 |
| | 0.16 | 2.324 | 3.025 | 2.412 | 2.326 | 3.046 | 2.412 |
| | 0.18 | 2.298 | 3.022 | 2.393 | 2.297 | 3.062 | 2.388 |
| | 0.22 | 2.335 | 3.022 | 2.426 | 2.330 | 3.125 | 2.415 |
| 0.60 | 0.00 | 2.414 | 2.977 | 2.476 | 2.414 | 2.965 | 2.476 |
| | 0.12 | 2.300 | 2.935 | 2.353 | 2.301 | 2.922 | 2.354 |
| | 0.15 | 2.241 | 2.907 | 2.353 | 2.240 | 2.931 | 2.354 |
| | 0.18 | 2.218 | 2.913 | 2.305 | 2.218 | 2.973 | 2.303 |
| | 0.20 | 2.247 | 2.915 | 2.331 | 2.244 | 3.012 | 2.324 |
| 0.63 | 0.00 | 2.264 | 2.844 | 2.330 | 2.264 | 2.827 | 2.336 |
| | 0.10 | 2.189 | 2.803 | 2.412 | 2.189 | 2.810 | 2.415 |
| | 0.12 | 2.174 | 2.804 | 2.251 | 2.174 | 2.825 | 2.249 |
| | 0.14 | 2.160 | 2.802 | 2.234 | 2.160 | 2.837 | 2.237 |
| | 0.16 | 2.148 | 2.803 | 2.221 | 2.148 | 2.865 | 2.222 |
| | 0.20 | 2.214 | 2.819 | 2.293 | 2.211 | 2.946 | 2.284 |
| 0.66 | 0.00 | 2.131 | 2.740 | 2.195 | 2.131 | 2.705 | 2.193 |
| | 0.10 | 2.098 | 2.705 | 2.169 | 2.098 | 2.732 | 2.169 |
| | 0.12 | 2.092 | 2.707 | 2.160 | 2.092 | 2.748 | 2.164 |
| | 0.14 | 2.084 | 2.707 | 2.158 | 2.085 | 2.766 | 2.158 |
| | 0.16 | 2.101 | 2.714 | 2.180 | 2.101 | 2.799 | 2.183 |
| | 0.18 | 2.143 | 2.728 | 2.221 | 2.150 | 2.838 | 2.222 |
| 0.69 | 0.00 | 2.023 | 2.619 | 2.093 | 2.023 | 2.628 | 2.094 |
| | 0.10 | 2.028 | 2.622 | 2.108 | 2.028 | 2.657 | 2.092 |
| | 0.12 | 2.033 | 2.624 | 2.101 | 2.032 | 2.680 | 2.108 |
| | 0.14 | 2.055 | 2.628 | 2.131 | 2.055 | 2.699 | 2.131 |
| | 0.16 | 2.102 | 2.646 | 2.183 | 2.106 | 2.736 | 2.184 |
| | 0.18 | 2.204 | 2.670 | 2.230 | 2.205 | 2.777 | 2.236 |
| 0.72 | 0.00 | 1.964 | 2.529 | 2.027 | 1.964 | 2.559 | 2.028 |
| | 0.10 | 2.016 | 2.545 | 2.083 | 2.015 | 2.601 | 2.074 |
| | 0.12 | 2.039 | 2.562 | 2.105 | 2.041 | 2.622 | 2.104 |
| | 0.14 | 2.073 | 2.576 | 2.146 | 2.084 | 2.654 | 2.153 |
| | 0.16 | 2.129 | 2.608 | 2.206 | 2.148 | 2.694 | 2.206 |

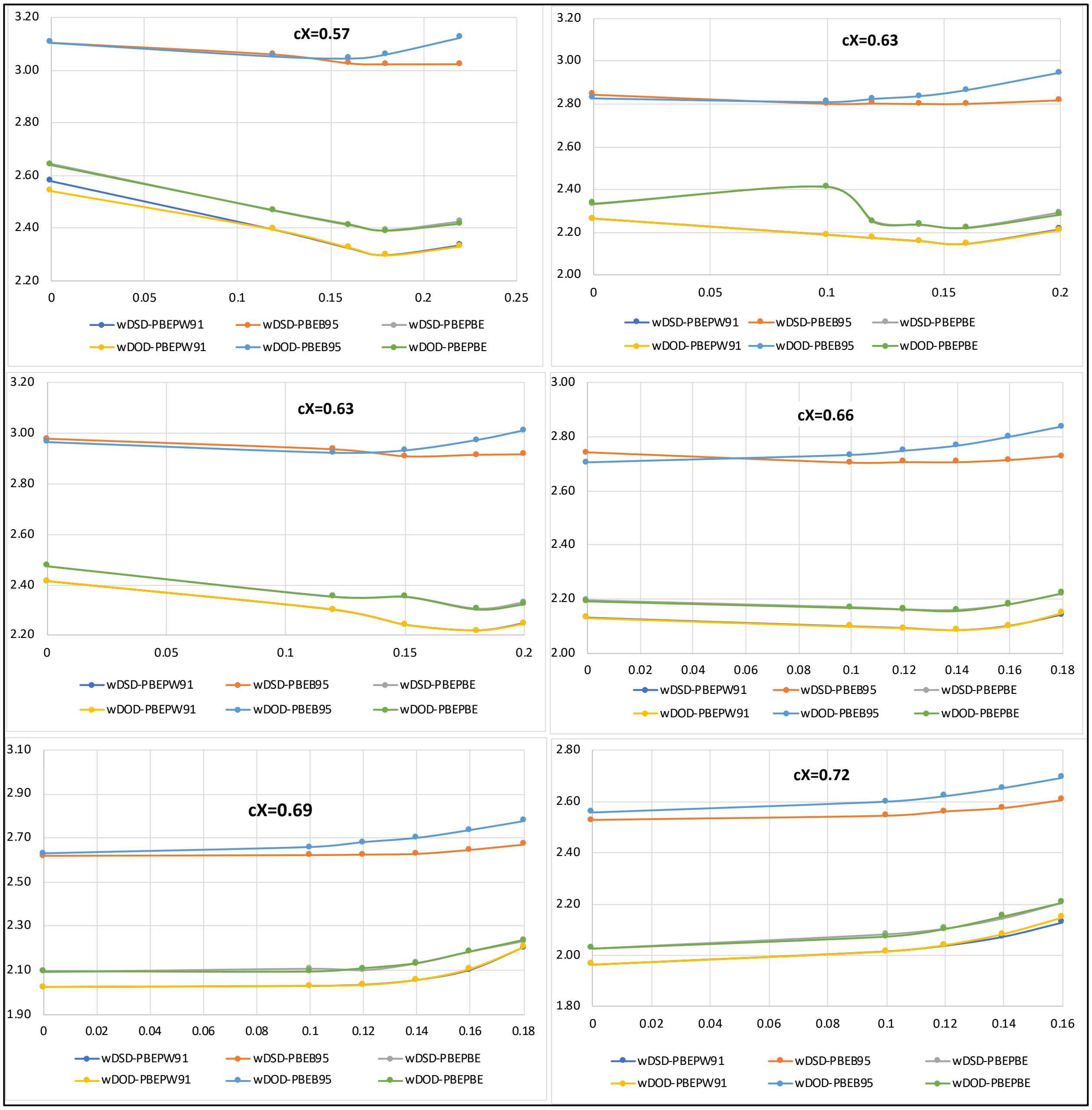


**Figure S1: Change of WTMAD2(kcal/mol) for ω$DSD_X$ and ω$DOD_X$-XC-D3BJ [XC=PBEB95, PBEPBE, PBEPW91] with respect to range separation parameter ω (x axis) for different cX,HF.**

## SI.4. Division of WTMAD2 into five major subcategories:

Table S4: Division of total WTMAD2(kcal/mol) into five major subsets: Small Molecule Thermochemistry (THERMO), Intermolecular Interactions (INTERMOL), Conformers/Intramolecular Interactions (CONF), Barrier Heights (BARRIERS), Small Molecule Thermochemistry (THERMO) and Large-species Reaction Energies (LARGE)

| Functionals | THERMO | BARRIERS | LARGE | CONF | INTERMOL | WTMAD2 |
|---|---|---|---|---|---|---|
| $xDSD_{75}$-PBEP86-D4 | 0.508 | 0.252 | 0.493 | 0.409 | 0.457 | 2.119 |
| $xDSD_{69}$-PBEP86-D4 | 0.550 | 0.277 | 0.502 | 0.425 | 0.532 | 2.285 |
| $xDSD_{74}$-PBEB95-D4 | 0.569 | 0.246 | 0.392 | 0.706 | 0.490 | 2.403 |
| $xDSD_{77}$-BLYP-D4 | 0.495 | 0.291 | 0.479 | 0.452 | 0.530 | 2.242 |
| $xDSD_{69}$-SCAN-D4 | 0.570 | 0.314 | 0.541 | 0.420 | 0.534 | 2.378 |
| $xDSD_{72}$-PBEPW91-D4 | 0.580 | 0.270 | 0.471 | 0.404 | 0.478 | 2.203 |
| $xDSD_{72}$-PBEPBE-D4 | 0.594 | 0.271 | 0.475 | 0.413 | 0.486 | 2.238 |
| $xDOD_{72}$-PBEP86-D4 | 0.572 | 0.234 | 0.512 | 0.408 | 0.470 | 2.196 |
| $xDOD_{69}$-PBEB95-D4 | 0.608 | 0.215 | 0.377 | 0.774 | 0.518 | 2.491 |
| $xDOD_{74}$-BLYP-D4 | 0.676 | 0.291 | 0.520 | 0.480 | 0.577 | 2.543 |
| $xDOD_{69}$-SCAN-D4 | 0.582 | 0.308 | 0.542 | 0.422 | 0.532 | 2.385 |
| $xDOD_{69}$-PBEPW91-D4 | 0.604 | 0.244 | 0.473 | 0.414 | 0.484 | 2.219 |
| $xDOD_{69}$-PBEPBE-D4 | 0.620 | 0.249 | 0.465 | 0.421 | 0.488 | 2.243 |
| $\omega DSD_{72}$-PBEP86-D4($\omega$=0.13) | 0.536 | 0.244 | 0.415 | 0.419 | 0.470 | 2.083 |
| $\omega DSD_{69}$-PBEP86-D4($\omega$=0.16) | 0.552 | 0.238 | 0.391 | 0.427 | 0.481 | 2.089 |
| $\omega DSD_{66}$-PBEP86-D4($\omega$=0.18) | 0.573 | 0.241 | 0.377 | 0.438 | 0.488 | 2.116 |
| $\omega DSD_{63}$-PBEP86-D4($\omega$=0.20) | 0.596 | 0.242 | 0.376 | 0.446 | 0.494 | 2.154 |
| $\omega DSD_{60}$-PBEP86-D4($\omega$=0.22) | 0.614 | 0.245 | 0.384 | 0.453 | 0.506 | 2.202 |
| $\omega DSD_{57}$-PBEP86-D4($\omega$=0.22) | 0.649 | 0.259 | 0.381 | 0.466 | 0.503 | 2.258 |
| $\omega DOD_{72}$-PBEP86-D4($\omega$=0.08) | 0.580 | 0.237 | 0.475 | 0.415 | 0.478 | 2.185 |
| $\omega DOD_{69}$-PBEP86-D4($\omega$=0.10) | 0.587 | 0.227 | 0.459 | 0.423 | 0.480 | 2.175 |
| $\omega DOD_{66}$-PBEP86-D4($\omega$=0.15) | 0.600 | 0.228 | 0.414 | 0.437 | 0.498 | 2.176 |
| $\omega DOD_{63}$-PBEP86-D4($\omega$=0.16) | 0.615 | 0.232 | 0.413 | 0.451 | 0.488 | 2.199 |
| $\omega DOD_{60}$-PBEP86-D4($\omega$=0.18) | 0.636 | 0.241 | 0.397 | 0.461 | 0.507 | 2.241 |
| $\omega DOD_{57}$-PBEP86-D4($\omega$=0.20) | 0.651 | 0.253 | 0.399 | 0.466 | 0.532 | 2.302 |
| ωB97M(2) | 0.430 | 0.214 | 0.418 | 0.577 | 0.492 | 2.131 |

# SI.5. MAD, MSD, RMSD and division of WTMAD2 into 55 subsets

Table S5: MAD, MSD and RMSD as well as breakdown of total WTMAD2 by each subset for ωDSD$_{72}$-PBEP86-D4 (ω=0.13)

| subs.name | MAD | MSD | RSMD | dWTMAD2 | 5MAD/4RMSD |
|---|---|---|---|---|---|
| ACONF | 0.039 | 0.036 | 0.052 | 0.012 | 0.9247 |
| ADIM6 | 0.373 | -0.373 | 0.41 | 0.0253 | 1.1371 |
| AHB21 | 0.204 | -0.116 | 0.305 | 0.0072 | 0.8385 |
| AL2X6 | 1.26 | -1.26 | 1.279 | 0.008 | 1.2322 |
| ALK8 | 2.093 | -1.68 | 2.769 | 0.0101 | 0.9449 |
| ALKBDE10 | 3.153 | -1.962 | 3.7 | 0.0119 | 1.065 |
| AMINO20X4 | 0.117 | -0.044 | 0.15 | 0.1458 | 0.9773 |
| BH76RC | 0.705 | 0.217 | 1.007 | 0.0375 | 0.8753 |
| BH76 | 0.862 | 0.613 | 1.816 | 0.1335 | 0.5933 |
| BHDIV10 | 0.581 | -0.133 | 0.744 | 0.0049 | 0.9768 |
| BHPERI | 0.639 | -0.494 | 0.753 | 0.0302 | 1.0602 |
| BHROT27 | 0.097 | 0.071 | 0.131 | 0.0159 | 0.9272 |
| BSR36 | 0.421 | -0.405 | 0.444 | 0.0355 | 1.1861 |
| BUT14DIOL | 0.06 | 0.06 | 0.067 | 0.052 | 1.111 |
| C60ISO | 4.904 | -4.717 | 6.792 | 0.017 | 0.9026 |
| CARBHB12 | 0.259 | 0.259 | 0.344 | 0.0195 | 0.9424 |
| CDIE20 | 0.261 | 0.254 | 0.355 | 0.0488 | 0.9197 |
| CHB6 | 0.757 | -0.729 | 0.959 | 0.0064 | 0.9859 |
| DARC | 0.539 | -0.339 | 0.577 | 0.0088 | 1.1683 |
| DC13 | 1.791 | 0.907 | 2.347 | 0.0161 | 0.9541 |
| DIPCS10 | 5.202 | -5.202 | 5.427 | 0.003 | 1.1981 |
| FH51 | 0.679 | -0.052 | 0.912 | 0.0423 | 0.9304 |
| G21EA | 2.837 | -2.707 | 3.26 | 0.08 | 1.0877 |
| G21IP | 2.275 | -1.505 | 2.679 | 0.0121 | 1.0611 |
| G2RC | 1.472 | 0.433 | 1.928 | 0.0272 | 0.9542 |
| HAL59 | 0.24 | 0.142 | 0.324 | 0.1171 | 0.9267 |
| HEAVY28 | 0.106 | 0.003 | 0.146 | 0.0906 | 0.9078 |
| HEAVYSB11 | 1.433 | -1.433 | 1.579 | 0.0103 | 1.1345 |
| ICONF | 0.115 | -0.027 | 0.151 | 0.0227 | 0.9505 |
| IDISP | 0.879 | -0.035 | 1.114 | 0.0141 | 0.9867 |
| IL16 | 0.264 | 0.264 | 0.314 | 0.0015 | 1.0509 |
| INV24 | 0.762 | 0.103 | 1.278 | 0.0218 | 0.7455 |
| ISO34 | 0.381 | -0.169 | 0.514 | 0.0337 | 0.9269 |
| ISOL24 | 0.748 | -0.063 | 1.06 | 0.031 | 0.8814 |
| MB16-43 | 9.915 | -9.524 | 11.277 | 0.0345 | 1.099 |
| MCONF | 0.097 | 0.055 | 0.118 | 0.0377 | 1.0298 |
| NBPRC | 0.288 | -0.065 | 0.409 | 0.0027 | 0.8808 |
| PA26 | 1.29 | 1.289 | 1.617 | 0.0067 | 0.9971 |
| PAREL | 0.358 | 0.045 | 0.573 | 0.0586 | 0.7817 |
| PCONF21 | 0.12 | -0.024 | 0.145 | 0.0505 | 1.0347 |
| PNICO23 | 0.072 | 0.022 | 0.09 | 0.0147 | 0.9982 |
| PX13 | 1.374 | -1.374 | 1.491 | 0.0203 | 1.1523 |
| RC21 | 1.622 | -1.366 | 2.101 | 0.0362 | 0.9649 |
| RG18 | 0.082 | -0.055 | 0.109 | 0.096 | 0.9351 |
| RSE43 | 0.686 | 0.664 | 1.29 | 0.1471 | 0.6647 |
| S22 | 0.103 | 0.018 | 0.129 | 0.0118 | 1.0044 |
| S66 | 0.164 | -0.037 | 0.206 | 0.075 | 0.9949 |
| SCONF | 0.076 | 0.008 | 0.114 | 0.0106 | 0.8319 |
| SIE4X4 | 4.312 | 4.312 | 4.973 | 0.0776 | 1.0838 |
| TAUT15 | 0.567 | -0.267 | 0.635 | 0.1059 | 1.1159 |
| UPU23 | 0.483 | 0.376 | 0.592 | 0.0735 | 1.0188 |
| W4-11 | 2.157 | -0.716 | 2.964 | 0.0373 | 0.9099 |
| WATER27 | 0.373 | 0.223 | 0.479 | 0.0047 | 0.9731 |
| WCPT18 | 0.865 | -0.617 | 1.044 | 0.0169 | 1.0362 |
| YBDE18 | 0.809 | 0.71 | 1.281 | 0.0112 | 0.789 |
| **GMTKN55** | | | | **2.0833** | |

Table S6: MAD, MSD and RMSD as well as breakdown of total WTMAD2 by each subset for ωDSD$_{72}$-PBEP86-D3BJ (ω=0.13)

| subs.name | MAD | MSD | RSMD | dWTMAD2 | 5MAD/4RMSD |
|---|---|---|---|---|---|
| ACONF | 0.043 | 0.041 | 0.056 | 0.0133 | 0.9601 |
| ADIM6 | 0.336 | -0.336 | 0.355 | 0.0228 | 1.1839 |
| AHB21 | 0.234 | -0.125 | 0.333 | 0.0083 | 0.878 |
| AL2X6 | 0.574 | -0.536 | 0.625 | 0.0036 | 1.1466 |
| ALK8 | 1.385 | -0.143 | 1.749 | 0.0067 | 0.9897 |
| ALKBDE10 | 3.103 | -1.872 | 3.54 | 0.0117 | 1.0957 |
| AMINO20X4 | 0.113 | -0.041 | 0.149 | 0.1404 | 0.9504 |
| BH76RC | 0.664 | 0.191 | 0.976 | 0.0353 | 0.8506 |
| BH76 | 0.84 | 0.589 | 1.78 | 0.13 | 0.5895 |
| BHDIV10 | 0.646 | -0.215 | 0.794 | 0.0054 | 1.0167 |
| BHPERI | 0.657 | -0.525 | 0.773 | 0.031 | 1.0624 |
| BHROT27 | 0.113 | 0.093 | 0.156 | 0.0185 | 0.9085 |
| BSR36 | 0.715 | -0.715 | 0.734 | 0.0603 | 1.2175 |
| BUT14DIOL | 0.028 | 0.011 | 0.036 | 0.0244 | 0.964 |
| C60ISO | 4.503 | -3.927 | 6.097 | 0.0156 | 0.9231 |
| CARBHB12 | 0.304 | 0.304 | 0.393 | 0.0229 | 0.9677 |
| CDIE20 | 0.258 | 0.252 | 0.357 | 0.0483 | 0.9053 |
| CHB6 | 1.112 | -1.112 | 1.293 | 0.0094 | 1.0753 |
| DARC | 0.629 | -0.449 | 0.679 | 0.0103 | 1.1567 |
| DC13 | 1.835 | 0.512 | 2.228 | 0.0165 | 1.0298 |
| DIPCS10 | 5.197 | -5.197 | 5.45 | 0.003 | 1.1921 |
| FH51 | 0.663 | -0.143 | 0.936 | 0.0414 | 0.8864 |
| G21EA | 2.903 | -2.774 | 3.301 | 0.0818 | 1.0993 |
| G21IP | 2.225 | -1.451 | 2.642 | 0.0118 | 1.0528 |
| G2RC | 1.488 | 0.25 | 1.953 | 0.0275 | 0.9522 |
| HAL59 | 0.247 | 0.098 | 0.345 | 0.1203 | 0.8956 |
| HEAVY28 | 0.08 | 0.018 | 0.12 | 0.0688 | 0.8362 |
| HEAVYSB11 | 0.837 | -0.801 | 1.126 | 0.006 | 0.9293 |
| ICONF | 0.103 | 0.009 | 0.129 | 0.0203 | 0.9986 |
| IDISP | 0.825 | -0.015 | 1.17 | 0.0132 | 0.8813 |
| IL16 | 0.465 | 0.465 | 0.525 | 0.0026 | 1.1087 |
| INV24 | 0.708 | 0.326 | 1.268 | 0.0202 | 0.6981 |
| ISO34 | 0.376 | -0.124 | 0.497 | 0.0333 | 0.9465 |
| ISOL24 | 0.691 | 0.008 | 0.993 | 0.0287 | 0.8706 |
| MB16-43 | 6.691 | -5.289 | 7.736 | 0.0233 | 1.0812 |
| MCONF | 0.226 | 0.19 | 0.252 | 0.0877 | 1.1199 |
| NBPRC | 0.362 | -0.223 | 0.437 | 0.0033 | 1.0362 |
| PA26 | 1.538 | 1.538 | 1.841 | 0.008 | 1.0443 |
| PAREL | 0.354 | 0.06 | 0.557 | 0.058 | 0.7941 |
| PCONF21 | 0.181 | 0.016 | 0.205 | 0.0761 | 1.1 |
| PNICO23 | 0.092 | 0.029 | 0.117 | 0.0187 | 0.9807 |
| PX13 | 1.472 | -1.472 | 1.588 | 0.0218 | 1.1593 |
| RC21 | 1.554 | -1.169 | 1.969 | 0.0347 | 0.9867 |
| RG18 | 0.095 | -0.072 | 0.13 | 0.1122 | 0.9175 |
| RSE43 | 0.658 | 0.636 | 1.256 | 0.1411 | 0.6546 |
| S22 | 0.132 | -0.028 | 0.18 | 0.0151 | 0.9173 |
| S66 | 0.148 | -0.052 | 0.178 | 0.0676 | 1.0354 |
| SCONF | 0.067 | 0.007 | 0.102 | 0.0094 | 0.8205 |
| SIE4X4 | 4.282 | 4.282 | 4.93 | 0.077 | 1.0856 |
| TAUT15 | 0.54 | -0.249 | 0.614 | 0.1009 | 1.1008 |
| UPU23 | 0.436 | 0.255 | 0.556 | 0.0664 | 0.9798 |
| W4-11 | 2.042 | -0.661 | 2.842 | 0.0353 | 0.898 |
| WATER27 | 0.511 | 0.383 | 0.631 | 0.0064 | 1.0119 |
| WCPT18 | 0.926 | -0.69 | 1.123 | 0.0181 | 1.0303 |
| YBDE18 | 0.921 | 0.858 | 1.338 | 0.0128 | 0.8608 |
| **GMTKN55** | | | | **2.1078** | |

Table S7: MAD, MSD and RMSD as well as breakdown of total WTMAD2 by each subset for ωDSD$_{69}$-PBEP86-D4 (ω=0.16)

| **subs.name** | **MAD** | **MSD** | **RSMD** | **dWTMAD2** | **5MAD/4RMSD** |
|---|---|---|---|---|---|
| ACONF | 0.032 | 0.027 | 0.043 | 0.0101 | 0.9443 |
| ADIM6 | 0.375 | -0.375 | 0.412 | 0.0254 | 1.1396 |
| AHB21 | 0.223 | -0.076 | 0.305 | 0.0079 | 0.9145 |
| AL2X6 | 1.182 | -1.182 | 1.211 | 0.0075 | 1.2207 |
| ALK8 | 2.064 | -1.673 | 2.771 | 0.01 | 0.9309 |
| ALKBDE10 | 3.267 | -1.903 | 3.811 | 0.0123 | 1.0714 |
| AMINO20X4 | 0.117 | -0.045 | 0.15 | 0.1459 | 0.9804 |
| BH76RC | 0.734 | 0.227 | 1.045 | 0.0391 | 0.878 |
| BH76 | 0.792 | 0.45 | 1.626 | 0.1227 | 0.6092 |
| BHDIV10 | 0.563 | -0.202 | 0.745 | 0.0047 | 0.9454 |
| BHPERI | 0.586 | -0.381 | 0.682 | 0.0277 | 1.0738 |
| BHROT27 | 0.11 | 0.065 | 0.143 | 0.0179 | 0.9589 |
| BSR36 | 0.392 | -0.364 | 0.422 | 0.0331 | 1.1626 |
| BUT14DIOL | 0.063 | 0.063 | 0.07 | 0.0547 | 1.1302 |
| C60ISO | 4.069 | -3.553 | 5.499 | 0.0141 | 0.9249 |
| CARBHB12 | 0.25 | 0.248 | 0.335 | 0.0188 | 0.9318 |
| CDIE20 | 0.239 | 0.217 | 0.352 | 0.0447 | 0.8477 |
| CHB6 | 0.777 | -0.743 | 0.976 | 0.0066 | 0.9952 |
| DARC | 0.602 | -0.477 | 0.669 | 0.0098 | 1.1239 |
| DC13 | 1.875 | 0.857 | 2.314 | 0.0168 | 1.0125 |
| DIPCS10 | 5.13 | -5.13 | 5.34 | 0.003 | 1.2008 |
| FH51 | 0.711 | -0.041 | 0.928 | 0.0443 | 0.9576 |
| G21EA | 2.766 | -2.651 | 3.199 | 0.078 | 1.0808 |
| G21IP | 2.264 | -1.472 | 2.675 | 0.012 | 1.0581 |
| G2RC | 1.584 | 0.473 | 2.046 | 0.0293 | 0.9681 |
| HAL59 | 0.246 | 0.137 | 0.327 | 0.12 | 0.9412 |
| HEAVY28 | 0.112 | 0 | 0.151 | 0.0955 | 0.9252 |
| HEAVYSB11 | 1.65 | -1.65 | 1.768 | 0.0119 | 1.1666 |
| ICONF | 0.116 | -0.024 | 0.152 | 0.0228 | 0.9507 |
| IDISP | 0.967 | -0.134 | 1.281 | 0.0155 | 0.9439 |
| IL16 | 0.301 | 0.301 | 0.346 | 0.0017 | 1.0874 |
| INV24 | 0.78 | 0.1 | 1.309 | 0.0223 | 0.7444 |
| ISO34 | 0.39 | -0.202 | 0.529 | 0.0345 | 0.9229 |
| ISOL24 | 0.728 | -0.061 | 1.005 | 0.0302 | 0.9059 |
| MB16-43 | 10.641 | -10.187 | 12.045 | 0.037 | 1.1043 |
| MCONF | 0.1 | 0.056 | 0.121 | 0.0388 | 1.0333 |
| NBPRC | 0.325 | -0.106 | 0.428 | 0.003 | 0.9483 |
| PA26 | 1.287 | 1.284 | 1.617 | 0.0067 | 0.9948 |
| PAREL | 0.357 | 0.035 | 0.574 | 0.0584 | 0.7765 |
| PCONF21 | 0.123 | -0.027 | 0.147 | 0.0517 | 1.0445 |
| PNICO23 | 0.068 | 0.026 | 0.089 | 0.0139 | 0.9561 |
| PX13 | 1.586 | -1.586 | 1.703 | 0.0234 | 1.164 |
| RC21 | 1.468 | -1.162 | 1.89 | 0.0327 | 0.9708 |
| RG18 | 0.086 | -0.065 | 0.119 | 0.1015 | 0.9056 |
| RSE43 | 0.604 | 0.568 | 1.154 | 0.1296 | 0.6543 |
| S22 | 0.103 | 0.002 | 0.128 | 0.0117 | 1.0019 |
| S66 | 0.16 | -0.048 | 0.204 | 0.073 | 0.9771 |
| SCONF | 0.084 | 0.007 | 0.127 | 0.0117 | 0.8214 |
| SIE4X4 | 4.759 | 4.759 | 5.506 | 0.0856 | 1.0803 |
| TAUT15 | 0.594 | -0.273 | 0.672 | 0.1109 | 1.1043 |
| UPU23 | 0.495 | 0.397 | 0.604 | 0.0755 | 1.0248 |
| W4-11 | 2.238 | -0.798 | 3.034 | 0.0387 | 0.922 |
| WATER27 | 0.389 | 0.081 | 0.474 | 0.0049 | 1.0258 |
| WCPT18 | 0.972 | -0.711 | 1.162 | 0.019 | 1.0456 |
| YBDE18 | 0.736 | 0.523 | 1.158 | 0.0102 | 0.7943 |
| **GMTKN55** | | | | **2.0888** | |

Table S8: MAD, MSD and RMSD as well as breakdown of total WTMAD2 by each subset for ωDSD$_{69}$-PBEP86-D3BJ (ω=0.16)

| subs.name | MAD | MSD | RSMD | dWTMAD2 | 5MAD/4RMSD |
|---|---|---|---|---|---|
| ACONF | 0.035 | 0.032 | 0.046 | 0.011 | 0.9573 |
| ADIM6 | 0.315 | -0.315 | 0.331 | 0.0213 | 1.188 |
| AHB21 | 0.258 | -0.074 | 0.332 | 0.0091 | 0.9725 |
| AL2X6 | 0.544 | -0.465 | 0.601 | 0.0035 | 1.1327 |
| ALK8 | 1.439 | -0.05 | 1.858 | 0.007 | 0.9685 |
| ALKBDE10 | 3.224 | -1.76 | 3.654 | 0.0121 | 1.1028 |
| AMINO20X4 | 0.115 | -0.042 | 0.15 | 0.1428 | 0.955 |
| BH76RC | 0.697 | 0.221 | 1.04 | 0.0371 | 0.8378 |
| BH76 | 0.771 | 0.409 | 1.592 | 0.1193 | 0.6053 |
| BHDIV10 | 0.67 | -0.309 | 0.828 | 0.0056 | 1.0119 |
| BHPERI | 0.632 | -0.477 | 0.735 | 0.0299 | 1.0747 |
| BHROT27 | 0.122 | 0.084 | 0.163 | 0.0198 | 0.9328 |
| BSR36 | 0.662 | -0.662 | 0.682 | 0.0558 | 1.2145 |
| BUT14DIOL | 0.03 | 0.009 | 0.037 | 0.0256 | 0.9889 |
| C60ISO | 3.822 | -2.904 | 5.003 | 0.0133 | 0.9548 |
| CARBHB12 | 0.297 | 0.297 | 0.386 | 0.0224 | 0.9616 |
| CDIE20 | 0.232 | 0.215 | 0.351 | 0.0435 | 0.827 |
| CHB6 | 1.126 | -1.126 | 1.315 | 0.0096 | 1.0709 |
| DARC | 0.655 | -0.498 | 0.717 | 0.0107 | 1.1426 |
| DC13 | 1.81 | 0.519 | 2.227 | 0.0162 | 1.0161 |
| DIPCS10 | 5.28 | -5.28 | 5.507 | 0.0031 | 1.1985 |
| FH51 | 0.693 | -0.107 | 0.944 | 0.0432 | 0.9179 |
| G21EA | 2.873 | -2.767 | 3.285 | 0.081 | 1.0932 |
| G21IP | 2.275 | -1.492 | 2.686 | 0.0121 | 1.059 |
| G2RC | 1.597 | 0.34 | 2.076 | 0.0295 | 0.9611 |
| HAL59 | 0.249 | 0.086 | 0.344 | 0.1211 | 0.9029 |
| HEAVY28 | 0.083 | 0.014 | 0.121 | 0.0713 | 0.8599 |
| HEAVYSB11 | 1.031 | -1.031 | 1.277 | 0.0074 | 1.0099 |
| ICONF | 0.104 | 0.014 | 0.131 | 0.0205 | 0.9944 |
| IDISP | 0.888 | -0.08 | 1.332 | 0.0142 | 0.8338 |
| IL16 | 0.508 | 0.508 | 0.568 | 0.0028 | 1.1178 |
| INV24 | 0.711 | 0.319 | 1.28 | 0.0203 | 0.6944 |
| ISO34 | 0.385 | -0.153 | 0.509 | 0.034 | 0.9453 |
| ISOL24 | 0.695 | -0.005 | 0.971 | 0.0289 | 0.8942 |
| MB16-43 | 7.208 | -5.874 | 8.262 | 0.0251 | 1.0905 |
| MCONF | 0.23 | 0.193 | 0.258 | 0.0896 | 1.1162 |
| NBPRC | 0.436 | -0.252 | 0.481 | 0.004 | 1.1344 |
| PA26 | 1.532 | 1.532 | 1.84 | 0.008 | 1.0404 |
| PAREL | 0.355 | 0.041 | 0.57 | 0.0581 | 0.777 |
| PCONF21 | 0.187 | 0.008 | 0.215 | 0.0789 | 1.0905 |
| PNICO23 | 0.093 | 0.029 | 0.121 | 0.019 | 0.9565 |
| PX13 | 1.713 | -1.713 | 1.826 | 0.0253 | 1.1727 |
| RC21 | 1.417 | -1.003 | 1.797 | 0.0316 | 0.986 |
| RG18 | 0.099 | -0.079 | 0.137 | 0.1169 | 0.9068 |
| RSE43 | 0.58 | 0.543 | 1.128 | 0.1244 | 0.6425 |
| S22 | 0.136 | -0.038 | 0.19 | 0.0155 | 0.8961 |
| S66 | 0.144 | -0.054 | 0.172 | 0.0658 | 1.0449 |
| SCONF | 0.075 | 0.009 | 0.117 | 0.0106 | 0.8065 |
| SIE4X4 | 4.758 | 4.758 | 5.485 | 0.0856 | 1.0844 |
| TAUT15 | 0.57 | -0.256 | 0.655 | 0.1064 | 1.0876 |
| UPU23 | 0.444 | 0.272 | 0.565 | 0.0677 | 0.9833 |
| W4-11 | 2.137 | -0.806 | 2.894 | 0.037 | 0.9233 |
| WATER27 | 0.447 | 0.257 | 0.559 | 0.0056 | 0.9984 |
| WCPT18 | 1.059 | -0.829 | 1.266 | 0.0207 | 1.0456 |
| YBDE18 | 0.824 | 0.657 | 1.216 | 0.0114 | 0.8474 |
| **GMTKN55** | | | | **2.1121** | |

Table S9: MAD, MSD and RMSD as well as breakdown of total WTMAD2 by each subset for ωDOD$_{72}$-PBEP86-D4 (ω=0.08)

| subs.name | MAD | MSD | RSMD | dWTMAD2 | 5MAD/4RMSD |
|---|---|---|---|---|---|
| ACONF | 0.033 | 0.027 | 0.042 | 0.0102 | 0.9664 |
| ADIM6 | 0.34 | -0.34 | 0.378 | 0.0231 | 1.1249 |
| AHB21 | 0.181 | -0.121 | 0.275 | 0.0064 | 0.823 |
| AL2X6 | 1.122 | -1.122 | 1.146 | 0.0071 | 1.2243 |
| ALK8 | 1.835 | -1.403 | 2.484 | 0.0089 | 0.9233 |
| ALKBDE10 | 2.892 | -2.444 | 3.532 | 0.0109 | 1.0235 |
| AMINO20X4 | 0.126 | -0.052 | 0.163 | 0.1562 | 0.9649 |
| BH76RC | 0.903 | 0.134 | 1.036 | 0.048 | 1.0897 |
| BH76 | 0.981 | 0.618 | 1.877 | 0.1519 | 0.6538 |
| BHDIV10 | 0.497 | 0.118 | 0.61 | 0.0042 | 1.0187 |
| BHPERI | 0.441 | 0.288 | 0.587 | 0.0208 | 0.9401 |
| BHROT27 | 0.073 | 0.054 | 0.098 | 0.012 | 0.9338 |
| BSR36 | 1.03 | -1.03 | 1.076 | 0.0868 | 1.1958 |
| BUT14DIOL | 0.052 | 0.05 | 0.062 | 0.045 | 1.0479 |
| C60ISO | 4.687 | -4.543 | 6.443 | 0.0163 | 0.9094 |
| CARBHB12 | 0.248 | 0.248 | 0.323 | 0.0187 | 0.9608 |
| CDIE20 | 0.247 | 0.217 | 0.335 | 0.0463 | 0.9225 |
| CHB6 | 0.808 | -0.775 | 1.004 | 0.0069 | 1.0053 |
| DARC | 0.343 | -0.206 | 0.382 | 0.0056 | 1.1237 |
| DC13 | 1.853 | 0.302 | 2.246 | 0.0166 | 1.0311 |
| DIPCS10 | 4.32 | -4.32 | 4.623 | 0.0025 | 1.1681 |
| FH51 | 0.738 | 0.035 | 0.945 | 0.046 | 0.9757 |
| G21EA | 2.742 | -2.44 | 3.192 | 0.0773 | 1.0737 |
| G21IP | 2.082 | -1.094 | 2.553 | 0.011 | 1.0196 |
| G2RC | 1.546 | 0.48 | 2.026 | 0.0286 | 0.9538 |
| HAL59 | 0.254 | 0.169 | 0.333 | 0.1237 | 0.9534 |
| HEAVY28 | 0.118 | 0.037 | 0.167 | 0.1006 | 0.8816 |
| HEAVYSB11 | 1.049 | -1.049 | 1.341 | 0.0075 | 0.9774 |
| ICONF | 0.124 | -0.059 | 0.162 | 0.0245 | 0.9598 |
| IDISP | 0.774 | 0.125 | 0.849 | 0.0124 | 1.1391 |
| IL16 | 0.27 | 0.27 | 0.319 | 0.0015 | 1.0586 |
| INV24 | 0.747 | 0.183 | 1.338 | 0.0214 | 0.6984 |
| ISO34 | 0.382 | -0.244 | 0.535 | 0.0338 | 0.8923 |
| ISOL24 | 0.789 | -0.212 | 1.135 | 0.0328 | 0.8687 |
| MB16-43 | 12.514 | -12.397 | 14.475 | 0.0436 | 1.0807 |
| MCONF | 0.073 | 0.022 | 0.088 | 0.0283 | 1.0345 |
| NBPRC | 0.326 | -0.124 | 0.519 | 0.003 | 0.7862 |
| PA26 | 1.747 | 1.747 | 2.023 | 0.0091 | 1.0789 |
| PAREL | 0.328 | 0.023 | 0.551 | 0.0538 | 0.7448 |
| PCONF21 | 0.12 | -0.014 | 0.144 | 0.0507 | 1.0462 |
| PNICO23 | 0.09 | 0.061 | 0.118 | 0.0183 | 0.9542 |
| PX13 | 0.957 | -0.932 | 1.082 | 0.0141 | 1.1061 |
| RC21 | 1.492 | -1.271 | 1.891 | 0.0333 | 0.9859 |
| RG18 | 0.075 | -0.04 | 0.096 | 0.0878 | 0.9728 |
| RSE43 | 0.726 | 0.703 | 1.28 | 0.1558 | 0.7094 |
| S22 | 0.1 | 0.018 | 0.129 | 0.0115 | 0.9701 |
| S66 | 0.162 | -0.022 | 0.201 | 0.074 | 1.0044 |
| SCONF | 0.079 | -0.056 | 0.113 | 0.0111 | 0.8767 |
| SIE4X4 | 4.367 | 4.367 | 5.144 | 0.0786 | 1.0611 |
| TAUT15 | 0.698 | -0.417 | 0.78 | 0.1304 | 1.1195 |
| UPU23 | 0.503 | 0.399 | 0.613 | 0.0767 | 1.0261 |
| W4-11 | 2.873 | 0.404 | 3.744 | 0.0497 | 0.9594 |
| WATER27 | 0.4 | 0.315 | 0.512 | 0.005 | 0.9769 |
| WCPT18 | 0.664 | -0.133 | 0.783 | 0.013 | 1.0606 |
| YBDE18 | 0.828 | 0.791 | 1.204 | 0.0115 | 0.8599 |
| **GMTKN55** | | | | **2.1844** | |

Table S10: MAD, MSD and RMSD as well as breakdown of total WTMAD2 by each subset for ωDOD$_{72}$-PBEP86-D3BJ (ω=0.08)

| **subs.name** | **MAD** | **MSD** | **RSMD** | **dWTMAD2** | **5MAD/4RMSD** |
|---|---|---|---|---|---|
| ACONF | 0.032 | 0.027 | 0.041 | 0.0098 | 0.9727 |
| ADIM6 | 0.267 | -0.267 | 0.284 | 0.0181 | 1.1776 |
| AHB21 | 0.215 | -0.12 | 0.302 | 0.0076 | 0.8909 |
| AL2X6 | 0.411 | -0.272 | 0.441 | 0.0026 | 1.1639 |
| ALK8 | 1.448 | 0.414 | 1.879 | 0.007 | 0.9633 |
| ALKBDE10 | 2.681 | -2.12 | 3.193 | 0.0101 | 1.0495 |
| AMINO20X4 | 0.12 | -0.05 | 0.157 | 0.1498 | 0.9591 |
| BH76RC | 0.882 | 0.112 | 1.025 | 0.0469 | 1.0754 |
| BH76 | 0.966 | 0.576 | 1.862 | 0.1495 | 0.6484 |
| BHDIV10 | 0.5 | 0.027 | 0.612 | 0.0042 | 1.0198 |
| BHPERI | 0.442 | 0.288 | 0.587 | 0.0209 | 0.9412 |
| BHROT27 | 0.082 | 0.066 | 0.11 | 0.0134 | 0.9329 |
| BSR36 | 1.392 | -1.392 | 1.487 | 0.1174 | 1.1703 |
| BUT14DIOL | 0.033 | -0.007 | 0.041 | 0.0287 | 1.0178 |
| C60ISO | 4.115 | -3.707 | 5.672 | 0.0143 | 0.9067 |
| CARBHB12 | 0.3 | 0.3 | 0.373 | 0.0226 | 1.0067 |
| CDIE20 | 0.222 | 0.191 | 0.319 | 0.0416 | 0.8717 |
| CHB6 | 1.204 | -1.204 | 1.386 | 0.0102 | 1.0851 |
| DARC | 0.422 | -0.351 | 0.488 | 0.0069 | 1.0816 |
| DC13 | 2.004 | -0.242 | 2.488 | 0.018 | 1.0066 |
| DIPCS10 | 4.157 | -4.157 | 4.497 | 0.0024 | 1.1556 |
| FH51 | 0.743 | -0.031 | 0.961 | 0.0463 | 0.9666 |
| G21EA | 2.736 | -2.413 | 3.18 | 0.0771 | 1.0754 |
| G21IP | 1.999 | -0.943 | 2.493 | 0.0106 | 1.002 |
| G2RC | 1.59 | 0.36 | 2.104 | 0.0294 | 0.9451 |
| HAL59 | 0.254 | 0.125 | 0.347 | 0.1238 | 0.9156 |
| HEAVY28 | 0.096 | 0.059 | 0.147 | 0.0823 | 0.8211 |
| HEAVYSB11 | 0.838 | -0.218 | 0.988 | 0.006 | 1.0606 |
| ICONF | 0.098 | -0.024 | 0.125 | 0.0194 | 0.9814 |
| IDISP | 0.685 | 0.169 | 0.854 | 0.011 | 1.0032 |
| IL16 | 0.46 | 0.46 | 0.533 | 0.0026 | 1.079 |
| INV24 | 0.692 | 0.425 | 1.334 | 0.0198 | 0.6482 |
| ISO34 | 0.359 | -0.211 | 0.494 | 0.0317 | 0.9083 |
| ISOL24 | 0.732 | -0.132 | 1.043 | 0.0304 | 0.8775 |
| MB16-43 | 8.886 | -7.857 | 10.702 | 0.0309 | 1.0379 |
| MCONF | 0.187 | 0.153 | 0.21 | 0.0729 | 1.1146 |
| NBPRC | 0.347 | -0.321 | 0.547 | 0.0032 | 0.7932 |
| PA26 | 2.102 | 2.102 | 2.362 | 0.011 | 1.112 |
| PAREL | 0.307 | 0.015 | 0.533 | 0.0503 | 0.7212 |
| PCONF21 | 0.175 | 0.015 | 0.202 | 0.0736 | 1.081 |
| PNICO23 | 0.112 | 0.077 | 0.132 | 0.0229 | 1.0572 |
| PX13 | 1.054 | -1.043 | 1.183 | 0.0156 | 1.1137 |
| RC21 | 1.383 | -1.062 | 1.735 | 0.0308 | 0.9959 |
| RG18 | 0.084 | -0.052 | 0.112 | 0.0994 | 0.9411 |
| RSE43 | 0.715 | 0.693 | 1.27 | 0.1532 | 0.7033 |
| S22 | 0.135 | -0.021 | 0.186 | 0.0154 | 0.9063 |
| S66 | 0.138 | -0.024 | 0.166 | 0.0632 | 1.039 |
| SCONF | 0.08 | -0.059 | 0.109 | 0.0111 | 0.9104 |
| SIE4X4 | 4.399 | 4.399 | 5.17 | 0.0791 | 1.0637 |
| TAUT15 | 0.709 | -0.431 | 0.796 | 0.1324 | 1.113 |
| UPU23 | 0.455 | 0.284 | 0.579 | 0.0694 | 0.9834 |
| W4-11 | 3.238 | 1.188 | 4.126 | 0.056 | 0.9808 |
| WATER27 | 0.613 | 0.559 | 0.797 | 0.0077 | 0.9618 |
| WCPT18 | 0.716 | -0.203 | 0.84 | 0.014 | 1.0659 |
| YBDE18 | 1.133 | 1.122 | 1.396 | 0.0157 | 1.0144 |
| **GMTKN55** | | | | **2.2202** | |

Table S11: MAD, MSD and RMSD as well as breakdown of total WTMAD2 by each subset for ωDOD$_{69}$-PBEP86-D3BJ(ω=0.10)

| subs.name | MAD | MSD | RSMD | dWTMAD2 | 5MAD/4RMSD |
|---|---|---|---|---|---|
| ACONF | 0.028 | 0.023 | 1.833 | 0.0088 | 0.9902 |
| ADIM6 | 0.234 | -0.234 | 3.358 | 0.0159 | 1.1789 |
| AHB21 | 0.231 | -0.079 | 22.486 | 0.0082 | 0.9543 |
| AL2X6 | 0.393 | -0.231 | 35.883 | 0.0025 | 1.1348 |
| ALK8 | 1.536 | 0.519 | 62.601 | 0.0074 | 0.927 |
| ALKBDE10 | 2.917 | -2.365 | 100.69 | 0.011 | 1.0575 |
| AMINO20X4 | 0.121 | -0.05 | 2.439 | 0.151 | 0.9604 |
| BH76RC | 0.875 | 0.159 | 21.392 | 0.0465 | 1.0609 |
| BH76 | 0.872 | 0.298 | 18.614 | 0.1349 | 0.6766 |
| BHDIV10 | 0.548 | -0.125 | 45.333 | 0.0046 | 1.0247 |
| BHPERI | 0.406 | 0.255 | 20.873 | 0.0192 | 0.9416 |
| BHROT27 | 0.085 | 0.067 | 6.273 | 0.0139 | 0.9098 |
| BSR36 | 1.426 | -1.426 | 16.197 | 0.1202 | 1.1646 |
| BUT14DIOL | 0.036 | -0.006 | 2.8 | 0.031 | 0.9982 |
| C60ISO | 3.577 | -2.915 | 98.252 | 0.0124 | 0.9279 |
| CARBHB12 | 0.313 | 0.313 | 6.036 | 0.0236 | 1.0127 |
| CDIE20 | 0.23 | 0.194 | 4.055 | 0.0431 | 0.8552 |
| CHB6 | 1.21 | -1.21 | 26.785 | 0.0103 | 1.0819 |
| DARC | 0.36 | -0.159 | 32.471 | 0.0059 | 1.1134 |
| DC13 | 1.999 | -0.123 | 54.978 | 0.0179 | 1.0439 |
| DIPCS10 | 4.587 | -4.587 | 654.26 | 0.0027 | 1.1712 |
| FH51 | 0.78 | 0.041 | 31.011 | 0.0486 | 0.98 |
| G21EA | 2.796 | -2.574 | 33.624 | 0.0788 | 1.082 |
| G21IP | 2.128 | -1.187 | 257.61 | 0.0113 | 1.0358 |
| G2RC | 1.701 | 0.459 | 51.263 | 0.0315 | 0.9426 |
| HAL59 | 0.26 | 0.131 | 4.592 | 0.1265 | 0.9095 |
| HEAVY28 | 0.099 | 0.063 | 1.241 | 0.0849 | 0.8268 |
| HEAVYSB11 | 0.856 | -0.513 | 58.023 | 0.0062 | 1.0064 |
| ICONF | 0.098 | -0.02 | 3.266 | 0.0193 | 0.9791 |
| IDISP | 0.661 | 0.219 | 14.223 | 0.0106 | 1.0173 |
| IL16 | 0.504 | 0.504 | 109.045 | 0.0028 | 1.0897 |
| INV24 | 0.668 | 0.401 | 31.846 | 0.0191 | 0.6408 |
| ISO34 | 0.362 | -0.229 | 14.57 | 0.032 | 0.8778 |
| ISOL24 | 0.826 | -0.21 | 21.919 | 0.0343 | 0.8845 |
| MB16-43 | 9.285 | -8.45 | 468.394 | 0.0323 | 1.0528 |
| MCONF | 0.182 | 0.146 | 4.971 | 0.0707 | 1.1027 |
| NBPRC | 0.395 | -0.329 | 24.683 | 0.0036 | 0.8943 |
| PA26 | 2.091 | 2.091 | 189.054 | 0.0109 | 1.1024 |
| PAREL | 0.317 | 0.009 | 4.63 | 0.0519 | 0.7047 |
| PCONF21 | 0.179 | 0.004 | 1.622 | 0.0752 | 1.0926 |
| PNICO23 | 0.128 | 0.097 | 4.273 | 0.0261 | 1.0253 |
| PX13 | 1.253 | -1.253 | 33.362 | 0.0185 | 1.1404 |
| RC21 | 1.225 | -0.898 | 35.699 | 0.0273 | 1.0121 |
| RG18 | 0.084 | -0.051 | 0.58 | 0.0989 | 0.9408 |
| RSE43 | 0.604 | 0.567 | 7.602 | 0.1296 | 0.6977 |
| S22 | 0.134 | -0.024 | 7.302 | 0.0153 | 0.89 |
| S66 | 0.133 | -0.018 | 5.467 | 0.0607 | 1.039 |
| SCONF | 0.088 | -0.062 | 4.6 | 0.0123 | 0.9155 |
| SIE4X4 | 4.993 | 4.993 | 33.725 | 0.0898 | 1.0693 |
| TAUT15 | 0.713 | -0.426 | 3.046 | 0.1331 | 1.1036 |
| UPU23 | 0.46 | 0.295 | 5.723 | 0.07 | 0.9847 |
| W4-11 | 2.713 | 0.306 | 306.914 | 0.0469 | 0.9442 |
| WATER27 | 0.621 | 0.524 | 81.174 | 0.0078 | 0.9738 |
| WCPT18 | 0.793 | -0.376 | 34.988 | 0.0155 | 1.0474 |
| YBDE18 | 0.791 | 0.734 | 49.276 | 0.011 | 0.8884 |
| **GMTKN55** | | | | **2.2043** | |

Table S12: MAD, MSD and RMSD as well as breakdown of total WTMAD2 by each subset for ωDOD$_{69}$-PBEP86-D4(ω=0.10)

| subs.name | MAD | MSD | RSMD | dWTMAD2 | 5MAD/4RMSD |
|---|---|---|---|---|---|
| ACONF | 0.029 | 0.021 | 0.036 | 0.009 | 1.0042 |
| ADIM6 | 0.322 | -0.322 | 0.358 | 0.0218 | 1.1244 |
| AHB21 | 0.195 | -0.095 | 0.281 | 0.0069 | 0.8675 |
| AL2X6 | 1.15 | -1.15 | 1.17 | 0.0073 | 1.2287 |
| ALK8 | 1.855 | -1.462 | 2.554 | 0.009 | 0.908 |
| ALKBDE10 | 3.028 | -2.445 | 3.656 | 0.0114 | 1.0351 |
| AMINO20X4 | 0.123 | -0.052 | 0.16 | 0.1532 | 0.9639 |
| BH76RC | 0.905 | 0.169 | 1.053 | 0.0481 | 1.0746 |
| BH76 | 0.886 | 0.307 | 1.621 | 0.1371 | 0.6831 |
| BHDIV10 | 0.54 | -0.07 | 0.657 | 0.0045 | 1.0266 |
| BHPERI | 0.394 | 0.219 | 0.521 | 0.0186 | 0.947 |
| BHROT27 | 0.077 | 0.054 | 0.105 | 0.0126 | 0.9211 |
| BSR36 | 1.059 | -1.059 | 1.112 | 0.0893 | 1.1915 |
| BUT14DIOL | 0.062 | 0.062 | 0.071 | 0.0538 | 1.088 |
| C60ISO | 4.148 | -3.928 | 5.737 | 0.0144 | 0.9037 |
| CARBHB12 | 0.263 | 0.263 | 0.34 | 0.0198 | 0.9685 |
| CDIE20 | 0.26 | 0.227 | 0.357 | 0.0487 | 0.9115 |
| CHB6 | 0.805 | -0.75 | 0.985 | 0.0068 | 1.0216 |
| DARC | 0.313 | -0.064 | 0.366 | 0.0051 | 1.0681 |
| DC13 | 1.836 | 0.418 | 2.19 | 0.0165 | 1.0482 |
| DIPCS10 | 4.474 | -4.474 | 4.745 | 0.0026 | 1.1785 |
| FH51 | 0.784 | 0.099 | 0.994 | 0.0489 | 0.9854 |
| G21EA | 2.669 | -2.436 | 3.124 | 0.0753 | 1.0681 |
| G21IP | 2.127 | -1.19 | 2.567 | 0.0113 | 1.0358 |
| G2RC | 1.658 | 0.559 | 2.176 | 0.0307 | 0.9526 |
| HAL59 | 0.255 | 0.174 | 0.34 | 0.1244 | 0.9391 |
| HEAVY28 | 0.119 | 0.036 | 0.169 | 0.1019 | 0.8837 |
| HEAVYSB11 | 1.291 | -1.291 | 1.502 | 0.0093 | 1.0743 |
| ICONF | 0.121 | -0.056 | 0.159 | 0.0239 | 0.9492 |
| IDISP | 0.78 | 0.149 | 0.853 | 0.0125 | 1.1429 |
| IL16 | 0.292 | 0.292 | 0.338 | 0.0016 | 1.0802 |
| INV24 | 0.734 | 0.135 | 1.294 | 0.021 | 0.7089 |
| ISO34 | 0.389 | -0.268 | 0.562 | 0.0344 | 0.8653 |
| ISOL24 | 0.86 | -0.29 | 1.227 | 0.0357 | 0.8762 |
| MB16-43 | 13.04 | -13.018 | 14.972 | 0.0454 | 1.0887 |
| MCONF | 0.08 | 0.023 | 0.096 | 0.031 | 1.0367 |
| NBPRC | 0.336 | -0.133 | 0.509 | 0.0031 | 0.8239 |
| PA26 | 1.692 | 1.692 | 1.988 | 0.0088 | 1.064 |
| PAREL | 0.339 | 0.02 | 0.584 | 0.0555 | 0.7251 |
| PCONF21 | 0.119 | -0.021 | 0.142 | 0.0503 | 1.055 |
| PNICO23 | 0.089 | 0.073 | 0.117 | 0.0183 | 0.9537 |
| PX13 | 1.224 | -1.224 | 1.349 | 0.0181 | 1.1349 |
| RC21 | 1.313 | -1.08 | 1.658 | 0.0293 | 0.9905 |
| RG18 | 0.076 | -0.042 | 0.098 | 0.0893 | 0.9674 |
| RSE43 | 0.609 | 0.565 | 1.09 | 0.1307 | 0.6987 |
| S22 | 0.1 | 0.017 | 0.128 | 0.0114 | 0.9741 |
| S66 | 0.157 | -0.019 | 0.195 | 0.0717 | 1.0011 |
| SCONF | 0.086 | -0.054 | 0.126 | 0.012 | 0.8515 |
| SIE4X4 | 5.008 | 5.008 | 5.858 | 0.0901 | 1.0686 |
| TAUT15 | 0.697 | -0.405 | 0.784 | 0.1301 | 1.11 |
| UPU23 | 0.506 | 0.402 | 0.614 | 0.0771 | 1.0297 |
| W4-11 | 2.625 | 0.148 | 3.53 | 0.0454 | 0.9294 |
| WATER27 | 0.442 | 0.324 | 0.559 | 0.0056 | 0.9886 |
| WCPT18 | 0.752 | -0.365 | 0.904 | 0.0147 | 1.0406 |
| YBDE18 | 0.73 | 0.515 | 1.078 | 0.0101 | 0.8469 |
| **GMTKN55** | | | | **2.1752** | |

Table S13: MAD, MSD and RMSD as well as breakdown of total WTMAD2 by each subset for xDSD$_{75}$-PBEP86-D3BJ

| **subs.name** | **MAD** | **MSD** | **RSMD** | **dWTMAD2** | **5MAD/4RMSD** |
|---|---|---|---|---|---|
| ACONF | 0.06 | 0.059 | 0.075 | 0.0185 | 0.9952 |
| ADIM6 | 0.366 | -0.366 | 0.391 | 0.0248 | 1.1713 |
| AHB21 | 0.253 | -0.211 | 0.359 | 0.0089 | 0.8798 |
| AL2X6 | 0.71 | -0.71 | 0.758 | 0.0045 | 1.1703 |
| ALK8 | 1.027 | -0.019 | 1.18 | 0.005 | 1.0877 |
| ALKBDE10 | 2.751 | -2.192 | 3.325 | 0.0104 | 1.0342 |
| AMINO20X4 | 0.114 | -0.043 | 0.149 | 0.1417 | 0.9553 |
| BH76RC | 0.696 | 0.165 | 0.909 | 0.037 | 0.9579 |
| BH76 | 0.922 | 0.675 | 1.951 | 0.1427 | 0.5903 |
| BHDIV10 | 0.684 | -0.159 | 0.822 | 0.0057 | 1.0411 |
| BHPERI | 0.714 | -0.608 | 0.818 | 0.0337 | 1.0919 |
| BHROT27 | 0.103 | 0.1 | 0.138 | 0.0168 | 0.9358 |
| BSR36 | 0.995 | -0.995 | 1.036 | 0.0838 | 1.2005 |
| BUT14DIOL | 0.032 | 0.011 | 0.042 | 0.0277 | 0.9501 |
| C60ISO | 6.785 | -6.688 | 9.144 | 0.0236 | 0.9276 |
| CARBHB12 | 0.332 | 0.332 | 0.418 | 0.025 | 0.9921 |
| CDIE20 | 0.324 | 0.324 | 0.385 | 0.0607 | 1.0547 |
| CHB6 | 1.052 | -1.052 | 1.215 | 0.0089 | 1.0824 |
| DARC | 0.4 | 0.054 | 0.507 | 0.0065 | 0.9852 |
| DC13 | 1.912 | 0.623 | 2.425 | 0.0171 | 0.9856 |
| DIPCS10 | 4.825 | -4.825 | 5.099 | 0.0028 | 1.183 |
| FH51 | 0.631 | -0.085 | 0.894 | 0.0394 | 0.8823 |
| G21EA | 2.782 | -2.618 | 3.174 | 0.0784 | 1.0956 |
| G21IP | 2.106 | -1.31 | 2.523 | 0.0112 | 1.0433 |
| G2RC | 1.353 | 0.24 | 1.841 | 0.025 | 0.9187 |
| HAL59 | 0.251 | 0.135 | 0.357 | 0.1224 | 0.8801 |
| HEAVY28 | 0.083 | 0.049 | 0.126 | 0.0713 | 0.8285 |
| HEAVYSB11 | 0.818 | -0.472 | 1.016 | 0.0059 | 1.0072 |
| ICONF | 0.103 | -0.001 | 0.129 | 0.0204 | 1.0035 |
| IDISP | 0.614 | 0.246 | 0.741 | 0.0098 | 1.0365 |
| IL16 | 0.425 | 0.425 | 0.477 | 0.0024 | 1.1149 |
| INV24 | 0.666 | 0.255 | 1.174 | 0.019 | 0.7093 |
| ISO34 | 0.372 | -0.092 | 0.499 | 0.0329 | 0.9323 |
| ISOL24 | 0.962 | -0.113 | 1.397 | 0.0399 | 0.8608 |
| MB16-43 | 7.04 | -6.15 | 8.149 | 0.0245 | 1.08 |
| MCONF | 0.191 | 0.156 | 0.214 | 0.0743 | 1.1155 |
| NBPRC | 0.217 | -0.153 | 0.41 | 0.002 | 0.6609 |
| PA26 | 1.505 | 1.505 | 1.815 | 0.0078 | 1.0364 |
| PAREL | 0.349 | 0.076 | 0.566 | 0.0571 | 0.7692 |
| PCONF21 | 0.162 | 0.02 | 0.18 | 0.0684 | 1.1309 |
| PNICO23 | 0.098 | 0.058 | 0.12 | 0.0201 | 1.0207 |
| PX13 | 1.201 | -1.201 | 1.312 | 0.0177 | 1.1436 |
| RC21 | 1.697 | -1.385 | 2.127 | 0.0378 | 0.9972 |
| RG18 | 0.085 | -0.056 | 0.114 | 0.0998 | 0.9333 |
| RSE43 | 0.738 | 0.725 | 1.384 | 0.1583 | 0.6665 |
| S22 | 0.133 | -0.029 | 0.181 | 0.0152 | 0.9227 |
| S66 | 0.161 | -0.052 | 0.195 | 0.0735 | 1.0284 |
| SCONF | 0.05 | -0.011 | 0.078 | 0.0071 | 0.8054 |
| SIE4X4 | 3.813 | 3.813 | 4.403 | 0.0686 | 1.0824 |
| TAUT15 | 0.498 | -0.252 | 0.558 | 0.0929 | 1.114 |
| UPU23 | 0.423 | 0.245 | 0.54 | 0.0645 | 0.9798 |
| W4-11 | 2.02 | -0.694 | 2.941 | 0.0349 | 0.8583 |
| WATER27 | 0.696 | 0.641 | 0.897 | 0.0088 | 0.9706 |
| WCPT18 | 0.75 | -0.574 | 0.944 | 0.0146 | 0.9922 |
| YBDE18 | 0.951 | 0.937 | 1.399 | 0.0132 | 0.8497 |
| **GMTKN55** | | | | **2.1452** | |

Table S14: MAD, MSD and RMSD as well as breakdown of total WTMAD2 by each subset for xDSD$_{75}$-PBEP86-D4

| subs.name | MAD | MSD | RSMD | dWTMAD2 | 5MAD/4RMSD |
|---|---|---|---|---|---|
| ACONF | 0.053 | 0.052 | 0.068 | 0.0163 | 0.9657 |
| ADIM6 | 0.355 | -0.355 | 0.393 | 0.0241 | 1.1314 |
| AHB21 | 0.238 | -0.219 | 0.341 | 0.0084 | 0.8719 |
| AL2X6 | 1.41 | -1.41 | 1.424 | 0.0089 | 1.2376 |
| ALK8 | 1.825 | -1.399 | 2.306 | 0.0088 | 0.9893 |
| ALKBDE10 | 2.797 | -2.252 | 3.467 | 0.0105 | 1.0083 |
| AMINO20X4 | 0.117 | -0.042 | 0.15 | 0.1459 | 0.9791 |
| BH76RC | 0.716 | 0.181 | 0.937 | 0.0381 | 0.9549 |
| BH76 | 0.949 | 0.676 | 1.975 | 0.1469 | 0.6008 |
| BHDIV10 | 0.661 | -0.098 | 0.797 | 0.0055 | 1.037 |
| BHPERI | 0.724 | -0.617 | 0.829 | 0.0342 | 1.091 |
| BHROT27 | 0.087 | 0.083 | 0.119 | 0.0142 | 0.9174 |
| BSR36 | 0.767 | -0.767 | 0.79 | 0.0647 | 1.2134 |
| BUT14DIOL | 0.058 | 0.058 | 0.068 | 0.0507 | 1.0731 |
| C60ISO | 7.472 | -7.44 | 9.877 | 0.026 | 0.9457 |
| CARBHB12 | 0.305 | 0.305 | 0.386 | 0.023 | 0.9873 |
| CDIE20 | 0.33 | 0.33 | 0.388 | 0.0616 | 1.0628 |
| CHB6 | 0.716 | -0.678 | 0.891 | 0.0061 | 1.0046 |
| DARC | 0.372 | 0.119 | 0.487 | 0.0061 | 0.9545 |
| DC13 | 1.846 | 0.972 | 2.475 | 0.0165 | 0.9322 |
| DIPCS10 | 4.775 | -4.775 | 5.028 | 0.0028 | 1.1872 |
| FH51 | 0.646 | -0.032 | 0.879 | 0.0403 | 0.9193 |
| G21EA | 2.702 | -2.535 | 3.12 | 0.0762 | 1.0824 |
| G21IP | 2.123 | -1.327 | 2.539 | 0.0112 | 1.045 |
| G2RC | 1.337 | 0.352 | 1.811 | 0.0247 | 0.9228 |
| HAL59 | 0.228 | 0.142 | 0.317 | 0.1113 | 0.902 |
| HEAVY28 | 0.092 | 0.013 | 0.136 | 0.0787 | 0.8468 |
| HEAVYSB11 | 1.109 | -1.109 | 1.324 | 0.008 | 1.0468 |
| ICONF | 0.114 | -0.026 | 0.149 | 0.0224 | 0.9521 |
| IDISP | 0.659 | 0.239 | 0.786 | 0.0105 | 1.0468 |
| IL16 | 0.235 | 0.235 | 0.289 | 0.0013 | 1.0152 |
| INV24 | 0.697 | 0.065 | 1.173 | 0.0199 | 0.7426 |
| ISO34 | 0.383 | -0.128 | 0.516 | 0.0339 | 0.9282 |
| ISOL24 | 1.024 | -0.169 | 1.463 | 0.0425 | 0.8749 |
| MB16-43 | 10.462 | -10.178 | 11.709 | 0.0364 | 1.1169 |
| MCONF | 0.094 | 0.053 | 0.116 | 0.0367 | 1.0207 |
| NBPRC | 0.327 | -0.029 | 0.472 | 0.003 | 0.8671 |
| PA26 | 1.289 | 1.289 | 1.623 | 0.0067 | 0.9926 |
| PAREL | 0.358 | 0.066 | 0.585 | 0.0587 | 0.7653 |
| PCONF21 | 0.111 | -0.01 | 0.128 | 0.0468 | 1.0844 |
| PNICO23 | 0.077 | 0.03 | 0.091 | 0.0158 | 1.0618 |
| PX13 | 1.167 | -1.167 | 1.278 | 0.0172 | 1.1412 |
| RC21 | 1.767 | -1.523 | 2.231 | 0.0394 | 0.9899 |
| RG18 | 0.076 | -0.041 | 0.097 | 0.0892 | 0.9722 |
| RSE43 | 0.759 | 0.744 | 1.409 | 0.1627 | 0.673 |
| S22 | 0.113 | 0.045 | 0.141 | 0.0129 | 0.9994 |
| S66 | 0.17 | -0.009 | 0.207 | 0.0778 | 1.0264 |
| SCONF | 0.061 | -0.002 | 0.095 | 0.0085 | 0.8017 |
| SIE4X4 | 3.847 | 3.847 | 4.444 | 0.0692 | 1.082 |
| TAUT15 | 0.513 | -0.261 | 0.57 | 0.0958 | 1.125 |
| UPU23 | 0.466 | 0.351 | 0.577 | 0.0711 | 1.0094 |
| W4-11 | 2.066 | -0.675 | 2.966 | 0.0357 | 0.8708 |
| WATER27 | 0.689 | 0.643 | 0.875 | 0.0087 | 0.985 |
| WCPT18 | 0.725 | -0.556 | 0.912 | 0.0141 | 0.9945 |
| YBDE18 | 0.841 | 0.806 | 1.359 | 0.0117 | 0.774 |
| **GMTKN55** | | | | **2.1187** | |

Table S15: MAD, MSD and RMSD as well as breakdown of total WTMAD2 by each subset for $xDOD_{72}$-PBEP86-D3BJ

| subs.name | MAD | MSD | RSMD | dWTMAD2 | 5MAD/4RMSD |
|---|---|---|---|---|---|
| ACONF | 0.039 | 0.036 | 0.05 | 0.012 | 0.973 |
| ADIM6 | 0.267 | -0.267 | 0.286 | 0.0181 | 1.169 |
| AHB21 | 0.223 | -0.168 | 0.316 | 0.0079 | 0.8814 |
| AL2X6 | 0.456 | -0.361 | 0.48 | 0.0029 | 1.1865 |
| ALK8 | 1.236 | 0.548 | 1.593 | 0.006 | 0.9697 |
| ALKBDE10 | 2.742 | -2.564 | 3.317 | 0.0103 | 1.0334 |
| AMINO20X4 | 0.12 | -0.05 | 0.156 | 0.1492 | 0.9609 |
| BH76RC | 0.922 | 0.113 | 1.057 | 0.049 | 1.0902 |
| BH76 | 0.97 | 0.473 | 1.813 | 0.1502 | 0.6687 |
| BHDIV10 | 0.517 | -0.029 | 0.641 | 0.0043 | 1.0091 |
| BHPERI | 0.396 | 0.207 | 0.507 | 0.0187 | 0.9781 |
| BHROT27 | 0.086 | 0.074 | 0.109 | 0.014 | 0.9864 |
| BSR36 | 1.563 | -1.563 | 1.692 | 0.1317 | 1.1552 |
| BUT14DIOL | 0.036 | -0.004 | 0.045 | 0.031 | 0.9989 |
| C60ISO | 5.194 | -5.031 | 7.138 | 0.018 | 0.9095 |
| CARBHB12 | 0.33 | 0.33 | 0.402 | 0.0249 | 1.0257 |
| CDIE20 | 0.262 | 0.237 | 0.338 | 0.0491 | 0.9702 |
| CHB6 | 1.171 | -1.171 | 1.341 | 0.0099 | 1.0913 |
| DARC | 0.307 | 0.021 | 0.377 | 0.005 | 1.0177 |
| DC13 | 1.951 | -0.068 | 2.527 | 0.0175 | 0.965 |
| DIPCS10 | 4.039 | -4.039 | 4.393 | 0.0023 | 1.1493 |
| FH51 | 0.736 | 0.015 | 0.949 | 0.0459 | 0.9695 |
| G21EA | 2.685 | -2.367 | 3.105 | 0.0757 | 1.0809 |
| G21IP | 1.961 | -0.944 | 2.446 | 0.0104 | 1.0023 |
| G2RC | 1.568 | 0.364 | 2.145 | 0.029 | 0.9134 |
| HAL59 | 0.263 | 0.152 | 0.363 | 0.1282 | 0.9059 |
| HEAVY28 | 0.104 | 0.083 | 0.157 | 0.0889 | 0.8292 |
| HEAVYSB11 | 0.891 | -0.239 | 1.049 | 0.0064 | 1.0616 |
| ICONF | 0.097 | -0.025 | 0.124 | 0.0191 | 0.9781 |
| IDISP | 0.547 | 0.338 | 0.664 | 0.0087 | 1.0291 |
| IL16 | 0.456 | 0.456 | 0.522 | 0.0025 | 1.0903 |
| INV24 | 0.649 | 0.36 | 1.243 | 0.0186 | 0.6528 |
| ISO34 | 0.355 | -0.196 | 0.5 | 0.0314 | 0.8867 |
| ISOL24 | 0.937 | -0.238 | 1.351 | 0.0389 | 0.8672 |
| MB16-43 | 9.513 | -8.88 | 11.306 | 0.0331 | 1.0518 |
| MCONF | 0.167 | 0.133 | 0.188 | 0.0649 | 1.1066 |
| NBPRC | 0.326 | -0.297 | 0.54 | 0.003 | 0.7553 |
| PA26 | 2.03 | 2.03 | 2.305 | 0.0106 | 1.101 |
| PAREL | 0.31 | 0.03 | 0.548 | 0.0508 | 0.7071 |
| PCONF21 | 0.164 | 0.013 | 0.186 | 0.069 | 1.1018 |
| PNICO23 | 0.131 | 0.112 | 0.157 | 0.0268 | 1.0492 |
| PX13 | 1.029 | -1.027 | 1.155 | 0.0152 | 1.1137 |
| RC21 | 1.364 | -1.054 | 1.676 | 0.0304 | 1.0173 |
| RG18 | 0.08 | -0.045 | 0.105 | 0.0939 | 0.9482 |
| RSE43 | 0.692 | 0.667 | 1.229 | 0.1485 | 0.7043 |
| S22 | 0.135 | -0.017 | 0.188 | 0.0154 | 0.8995 |
| S66 | 0.143 | -0.016 | 0.173 | 0.0654 | 1.033 |
| SCONF | 0.083 | -0.069 | 0.11 | 0.0116 | 0.9399 |
| SIE4X4 | 4.394 | 4.394 | 5.171 | 0.079 | 1.0621 |
| TAUT15 | 0.669 | -0.418 | 0.753 | 0.125 | 1.1106 |
| UPU23 | 0.45 | 0.285 | 0.572 | 0.0685 | 0.9835 |
| W4-11 | 3.035 | 0.421 | 3.897 | 0.0525 | 0.9736 |
| WATER27 | 0.772 | 0.734 | 1.024 | 0.0097 | 0.9431 |
| WCPT18 | 0.693 | -0.255 | 0.811 | 0.0135 | 1.0682 |
| YBDE18 | 0.934 | 0.899 | 1.241 | 0.0129 | 0.9411 |
| **GMTKN55** | | | | **2.2359** | |

Table S16: MAD, MSD and RMSD as well as breakdown of total WTMAD2 by each subset for xDOD$_{72}$-PBEP86-D4

| subs.name | MAD | MSD | RSMD | dWTMAD2 | 5MAD/4RMSD |
|---|---|---|---|---|---|
| ACONF | 0.036 | 0.031 | 0.046 | 0.0111 | 0.9674 |
| ADIM6 | 0.307 | -0.307 | 0.343 | 0.0208 | 1.1173 |
| AHB21 | 0.203 | -0.174 | 0.295 | 0.0072 | 0.8619 |
| AL2X6 | 1.267 | -1.267 | 1.284 | 0.008 | 1.2334 |
| ALK8 | 1.704 | -1.284 | 2.246 | 0.0083 | 0.9482 |
| ALKBDE10 | 2.866 | -2.687 | 3.54 | 0.0108 | 1.0122 |
| AMINO20X4 | 0.123 | -0.051 | 0.159 | 0.1524 | 0.9644 |
| BH76RC | 0.927 | 0.14 | 1.066 | 0.0493 | 1.0875 |
| BH76 | 0.984 | 0.476 | 1.823 | 0.1524 | 0.6749 |
| BHDIV10 | 0.531 | 0.025 | 0.643 | 0.0044 | 1.0322 |
| BHPERI | 0.377 | 0.151 | 0.485 | 0.0178 | 0.9723 |
| BHROT27 | 0.072 | 0.058 | 0.093 | 0.0118 | 0.9733 |
| BSR36 | 1.238 | -1.238 | 1.32 | 0.1043 | 1.1718 |
| BUT14DIOL | 0.058 | 0.057 | 0.068 | 0.05 | 1.0585 |
| C60ISO | 6.095 | -6.035 | 8.122 | 0.0212 | 0.9381 |
| CARBHB12 | 0.284 | 0.284 | 0.357 | 0.0214 | 0.9974 |
| CDIE20 | 0.282 | 0.26 | 0.351 | 0.0528 | 1.0045 |
| CHB6 | 0.771 | -0.702 | 0.93 | 0.0065 | 1.0357 |
| DARC | 0.319 | 0.164 | 0.391 | 0.0052 | 1.0205 |
| DC13 | 1.913 | 0.461 | 2.356 | 0.0172 | 1.0153 |
| DIPCS10 | 4.098 | -4.098 | 4.403 | 0.0024 | 1.1633 |
| FH51 | 0.744 | 0.086 | 0.947 | 0.0464 | 0.9815 |
| G21EA | 2.631 | -2.313 | 3.069 | 0.0742 | 1.0717 |
| G21IP | 2.011 | -1.033 | 2.482 | 0.0107 | 1.0132 |
| G2RC | 1.533 | 0.497 | 2.073 | 0.0284 | 0.9246 |
| HAL59 | 0.244 | 0.166 | 0.326 | 0.119 | 0.9359 |
| HEAVY28 | 0.11 | 0.04 | 0.161 | 0.0944 | 0.8562 |
| HEAVYSB11 | 1.081 | -1.076 | 1.355 | 0.0078 | 0.9971 |
| ICONF | 0.118 | -0.056 | 0.156 | 0.0232 | 0.9424 |
| IDISP | 0.625 | 0.304 | 0.738 | 0.01 | 1.059 |
| IL16 | 0.253 | 0.25 | 0.301 | 0.0014 | 1.0517 |
| INV24 | 0.699 | 0.122 | 1.227 | 0.02 | 0.7127 |
| ISO34 | 0.38 | -0.231 | 0.545 | 0.0337 | 0.8725 |
| ISOL24 | 1.006 | -0.317 | 1.442 | 0.0418 | 0.8719 |
| MB16-43 | 13.689 | -13.64 | 15.487 | 0.0477 | 1.1048 |
| MCONF | 0.075 | 0.022 | 0.091 | 0.029 | 1.0291 |
| NBPRC | 0.35 | -0.109 | 0.557 | 0.0032 | 0.7859 |
| PA26 | 1.686 | 1.686 | 1.976 | 0.0088 | 1.067 |
| PAREL | 0.333 | 0.029 | 0.576 | 0.0546 | 0.7237 |
| PCONF21 | 0.109 | -0.014 | 0.132 | 0.0458 | 1.0303 |
| PNICO23 | 0.095 | 0.071 | 0.12 | 0.0194 | 0.9877 |
| PX13 | 1 | -0.992 | 1.121 | 0.0148 | 1.1151 |
| RC21 | 1.473 | -1.252 | 1.834 | 0.0329 | 1.0041 |
| RG18 | 0.072 | -0.033 | 0.091 | 0.085 | 0.9923 |
| RSE43 | 0.704 | 0.676 | 1.246 | 0.1509 | 0.706 |
| S22 | 0.108 | 0.041 | 0.137 | 0.0124 | 0.9863 |
| S66 | 0.163 | 0.003 | 0.199 | 0.0747 | 1.0222 |
| SCONF | 0.078 | -0.057 | 0.113 | 0.011 | 0.8646 |
| SIE4X4 | 4.415 | 4.415 | 5.194 | 0.0794 | 1.0625 |
| TAUT15 | 0.665 | -0.407 | 0.744 | 0.1241 | 1.1161 |
| UPU23 | 0.495 | 0.385 | 0.604 | 0.0754 | 1.0231 |
| W4-11 | 2.841 | 0.105 | 3.711 | 0.0491 | 0.957 |
| WATER27 | 0.647 | 0.611 | 0.84 | 0.0082 | 0.9637 |
| WCPT18 | 0.653 | -0.252 | 0.772 | 0.0127 | 1.0562 |
| YBDE18 | 0.78 | 0.661 | 1.184 | 0.0108 | 0.824 |
| **GMTKN55** | | | | **2.1957** | |

# SI.6. MAD for CHAL336 and four subsets.

Table S17: Mean Absolute Deviation (in kcal/mol) statistics of various DSD-DHs for the full CHAL336 dataset and it's four subset. [red (worst) via yellow to green (best)]

| Basis set | Functional | MAD (kcal/mol) | | | | |
|---|---|---|---|---|---|---|
| | | CHAL336 | Chalcogen-Chalcogen | Chalcogen-Nitrogen | Chalcogen-π | Chalcogen-Halogen |
| ma-def2-QZVPP | revDSD-PBEP86-D3BJ | 0.39 | 0.51 | 0.26 | 0.44 | 0.38 |
| | revDSD-PBEP86-D4 | 0.49 | 0.67 | 0.40 | 0.20 | 0.46 |
| | xDSD75-PBEP86-D3BJ | 0.34 | 0.44 | 0.30 | 0.23 | 0.32 |
| | xDSD75-PBEP86-D4 | 0.46 | 0.69 | 0.34 | 0.16 | 0.41 |
| | xDOD72-PBEP86-D3BJ | 0.33 | 0.41 | 0.29 | 0.26 | 0.32 |
| | xDOD72-PBEP86-D4 | 0.47 | 0.67 | 0.35 | 0.20 | 0.46 |
| | ωDSD72-PBEP86-D3BJ(ω=0.13) | 0.34 | 0.40 | 0.28 | 0.21 | 0.37 |
| | ωDSD72-PBEP86-D4(ω=0.13) | 0.45 | 0.57 | 0.46 | 0.18 | 0.39 |
| | ωDOD72-PBEP86-D3BJ(ω=0.08) | 0.33 | 0.38 | 0.28 | 0.23 | 0.34 |
| | ωDOD72-PBEP86-D4(ω=0.08) | 0.42 | 0.51 | 0.45 | 0.19 | 0.37 |
| | ωB97M(2) | 0.44 | 0.58 | 0.29 | 0.17 | 0.49 |
| ma-{T,Q} | revDSD-PBEP86-D3BJ | 0.41 | 0.59 | 0.22 | 0.52 | 0.37 |
| | revDSD-PBEP86-D4 | 0.34 | 0.44 | 0.33 | 0.21 | 0.30 |
| | xDSD75-PBEP86-D3BJ | 0.33 | 0.34 | 0.24 | 0.34 | 0.40 |
| | xDSD75-PBEP86-D4 | 0.28 | 0.32 | 0.29 | 0.11 | 0.28 |
| | xDOD72-PBEP86-D3BJ | 0.32 | 0.42 | 0.23 | 0.32 | 0.31 |
| | xDOD72-PBEP86-D4 | 0.29 | 0.35 | 0.29 | 0.14 | 0.28 |
| | ωDSD72-PBEP86-D3BJ(ω=0.13) | 0.30 | 0.37 | 0.23 | 0.32 | 0.28 |
| | ωDSD72-PBEP86-D4(ω=0.13) | 0.34 | 0.30 | 0.39 | 0.21 | 0.36 |
| | ωDOD72-PBEP86-D3BJ(ω=0.08) | 0.31 | 0.45 | 0.22 | 0.30 | 0.26 |
| | ωDOD72-PBEP86-D4(ω=0.08) | 0.33 | 0.31 | 0.37 | 0.20 | 0.36 |
| | ωB97M(2) | 0.35 | 0.32 | 0.26 | 0.22 | 0.46 |

## SI.7. **QCHEM sample inputs for CO:**

(a) **ωDSD$_{72}$-PBEP86-D3BJ(ω=0.13):**

```
$comment
wDSD72-PBEP86-D3BJ with w=0.13
$end

$rem
SYMMETRY                false
BASIS                   def2-QZVPP
XC_GRID                 3
ECP                     def2-ECP
N_FROZEN_CORE           FC
AUX_BASIS_CORR          rimp2-def2-QZVPPD
MAX_SCF_CYCLES          1000
SCF_CONVERGENCE         7
MEM_STATIC              2000
SET_ITER                100
MOLDEN_FORMAT           false
PRINT_ORBITALS          10
SCF_FINAL_PRINT         1
THRESH                  12
LRC_DFT                 TRUE
OMEGA                   130
combine_K               TRUE
exchange                gen
correlation             rimp2
DH                      TRUE
SSS_FACTOR              153700
SOS_FACTOR              673830
DFT_D                   D3_BJ
DFT_D3_S6               34760
DFT_D3_S8               0
DFT_D3_A2               550000
DFT_D3_A1               000000
$end

$xc_functional
 X HF   0.72
 X wPBE 0.28
 C P86  1.00
$end

$molecule
0 1
 C        0.000000000000      0.000000000000      0.000000000000
 O        0.000000000000      0.000000000000      1.131400000000
$end
```

(b) **ωDOD$_{69}$-PBEP86-D3BJ(ω=0.10):**

```
$comment
wDOD69-PBEP86-D3BJ with w=0.10
$end
```

```
$rem
SYMMETRY             false
BASIS                def2-QZVPP
XC_GRID              3
ECP                  def2-ECP
N_FROZEN_CORE        FC
AUX_BASIS_CORR       rimp2-def2-QZVPPD
MAX_SCF_CYCLES       1000
SCF_CONVERGENCE      7
MEM_STATIC           2000
SET_ITER             100
MOLDEN_FORMAT        false
PRINT_ORBITALS       10
SCF_FINAL_PRINT      1
THRESH               12
LRC_DFT              TRUE
OMEGA                100
combine_K            TRUE
exchange             gen
correlation          rimp2
DH                   TRUE
SSS_FACTOR           000000
SOS_FACTOR           702490
DFT_D                D3_BJ
DFT_D3_S6            42353
DFT_D3_S8            0
DFT_D3_A2            550000
DFT_D3_A1            000000
$end

$xc_functional
 X HF   0.72
 X wPBE 0.28
 C P86  1.00
$end

$molecule
0 1
 C        0.000000000000      0.000000000000      0.000000000000
 O        0.000000000000      0.000000000000      1.131400000000
$end
```

## ORCA sample inputs for CO:

**(a) $xDSD_{75}$-PBEP86-D3BJ:**

```
! PAL8
%maxcore 2000

! RKS def2-QZVPP def2-qzvpp/c def2/j tightscf rijcosx GRIDX9 GRID6 NoPop
XYZFILE
%method
   Exchange X_PBE
   Correlation C_P86
   ScalHFX 0.75
```

```
   ScalDFX 0.25
   ScalGGAC 1.0
   ScalLDAC 1.0
end
* xyz 0 1
 C       0.000000000000      0.000000000000      0.000000000000
 O       0.000000000000      0.000000000000      1.131400000000
*

$new_job
%maxcore 2000
! RKS def2-QZVPP def2-qzvpp/c def2/j tightscf rijcosx GRID6 GRIDX9 d3bj
%method
   Exchange X_PBE
   Correlation C_P86
   ScalHFX 0.75
   ScalDFX 0.25
   ScalGGAC 0.35900
   ScalLDAC 0.35900
   ScalMP2C 1.0
   D3S6 0.3071
   D3S8 0.0000
   D3A1 0.0000
   D3A2 5.6000
end
%mp2
   DoSCS true
   PS 0.67765
   PT 0.13886
end

%scf
 maxiter 1
 tole 10000
 tolg 10000
end

* xyzfile 0 1
```

**(b) xDOD$_{72}$-PBEP86-D3BJ:**

```
! PAL8
%maxcore 2000

! RKS def2-QZVPP def2-qzvpp/c def2/j tightscf rijcosx GRIDX9 GRID6 NoPop
XYZFILE
%method
   Exchange X_PBE
   Correlation C_P86
   ScalHFX 0.72
   ScalDFX 0.28
   ScalGGAC 1.0
   ScalLDAC 1.0
end
* xyz 0 1
```

```
 C        0.000000000000      0.000000000000      0.000000000000
 O        0.000000000000      0.000000000000      1.131400000000
*

$new_job
%maxcore 2000
! RKS def2-QZVPP def2-qzvpp/c def2/j tightscf rijcosx GRID6 GRIDX9 d3bj
%method
   Exchange X_PBE
   Correlation C_P86
   ScalHFX 0.72
   ScalDFX 0.28
   ScalGGAC 0.41075
   ScalLDAC 0.41075
   ScalMP2C 1.0
   D3S6 0.4278
   D3S8 0.0000
   D3A1 0.0000
   D3A2 5.6000
end
%mp2
   DoSCS true
   PS 0.66721
   PT 0.00000
end

%scf
 maxiter 1
 tole 10000
 tolg 10000
end

* xyzfile 0 1
```

# References:


(1) Goerigk, L.; Hansen, A.; Bauer, C.; Ehrlich, S.; Najibi, A.; Grimme, S. A Look at the Density Functional Theory Zoo with the Advanced GMTKN55 Database for General Main Group Thermochemistry, Kinetics and Noncovalent Interactions. *Phys. Chem. Chem. Phys.* **2017**, *19*, 32184–32215.

(2) Gruzman, D.; Karton, A.; Martin, J. M. L. Performance of Ab Initio and Density Functional Methods for Conformational Equilibria of C n H 2 n +2 Alkane Isomers ( n = 4−8) †. *J. Phys. Chem. A* **2009**, *113*, 11974–11983.

(3) Grimme, S.; Antony, J.; Ehrlich, S.; Krieg, H. A Consistent and Accurate Ab Initio Parametrization of Density Functional Dispersion Correction (DFT-D) for the 94 Elements H-Pu. *J. Chem. Phys.* **2010**, *132*, 154104.

(4) Lao, K. U.; Schäffer, R.; Jansen, G.; Herbert, J. M. Accurate Description of Intermolecular Interactions Involving Ions Using Symmetry-Adapted Perturbation Theory. *J. Chem. Theory Comput.* **2015**, *11*, 2473–2486.

(5) Yu, H.; Truhlar, D. G. Components of the Bond Energy in Polar Diatomic Molecules, Radicals, and Ions Formed by Group-1 and Group-2 Metal Atoms. *J. Chem. Theory Comput.* **2015**, *11*, 2968–2983.

(6) Kesharwani, M. K.; Karton, A.; Martin, J. M. L. Benchmark Ab Initio Conformational Energies for the Proteinogenic Amino Acids through Explicitly Correlated Methods. Assessment of Density Functional Methods. *J. Chem. Theory Comput.* **2016**, *12*, 444–454.

(7) Goerigk, L.; Grimme, S. A General Database for Main Group Thermochemistry, Kinetics, and Noncovalent Interactions − Assessment of Common and Reparameterized ( Meta - )GGA Density Functionals. *J. Chem. Theory Comput.* **2010**, *6*, 107–126.

(8) Zhao, Y.; Lynch, B. J.; Truhlar, D. G. Multi-Coefficient Extrapolated Density Functional Theory for Thermochemistry and Thermochemical Kinetics. *Phys. Chem. Chem. Phys.* **2005**, *7*, 43.

(9) Zhao, Y.; González-Garda, N.; Truhlar, D. G. Benchmark Database of Barrier Heights for Heavy Atom Transfer, Nucleophilic Substitution, Association, and Unimolecular Reactions and Its Use to Test Theoretical Methods. *J. Phys. Chem. A* **2005**, *109*, 2012–2018.

(10) Goerigk, L.; Grimme, S.; Chemie, T. O. A General Database for Main Group Thermochemistry , Kinetics , and Non-Covalent Interactions – Assessment of Common and Reparameterized ( Meta- ) GGA Density Functionals Supporting Information. **2009**, 1–32.

(11) Guner, V.; Khuong, K. S.; Leach, A. G.; Lee, P. S.; Bartberger, M. D.; Houk, K. N. A Standard Set of Pericyclic Reactions of Hydrocarbons for the Benchmarking of Computational Methods : The Performance of Ab Initio , Density Functional , CASSCF , CASPT2 , and CBS-QB3 Methods for the Prediction of Activation Barriers , Reaction Energetic. **2003**, 11445–11459.

(12) Ess, D. H.; Houk, K. N. Activation Energies of Pericyclic Reactions : Performance of DFT , MP2 , and CBS-QB3 Methods for the Prediction of Activation Barriers and Reaction Energetics of 1 , 3-Dipolar Cycloadditions , and Revised Activation Enthalpies for a Standard Set of Hydroc. **2005**, 9542–9553.

(13) Dinadayalane, T. C.; Vijaya, R.; Smitha, A.; Sastry, G. N. Diels−Alder Reactivity of Butadiene and Cyclic Five-Membered Dienes ((CH) 4 X, X = CH 2 , SiH 2 , O, NH, PH, and S) with Ethylene: A Benchmark Study. *J. Phys. Chem. A* **2002**, *106*, 1627–1633.

(14) Steinmann, S. N.; Csonka, G.; Corminboeuf, C. Unified Inter- and Intramolecular Dispersion Correction Functional Theory. *J. Chem. Theory Comput.* **2009**, *5*, 2950–2958.

(15) Krieg, H.; Grimme, S. Thermochemical Benchmarking of Hydrocarbon Bond Separation Reaction Energies: Jacob's Ladder Is Not Reversed! *Mol. Phys.* **2010**, *108*, 2655–2666.

(16) Kozuch, S.; Bachrach, S. M.; Martin, J. M. L. Conformational Equilibria in Butane-1,4-Diol: A Benchmark of a Prototypical System with Strong Intramolecular H - Bonds. **2014**.

(17) Sure, R.; Hansen, A.; Schwerdtfeger, P.; Grimme, S. Comprehensive Theoretical Study of All 1812 C60isomers. *Phys. Chem. Chem. Phys.* **2017**, *19*, 14296–14305.

(18) Yu, L.; Karton, A. Assessment of Theoretical Procedures for a Diverse Set of Isomerization Reactions Involving Double-Bond Migration in Conjugated Dienes. *Chem. Phys.* **2014**, *441*, 166–177.

(19) Johnson, E. R.; Mori-Sánchez, P.; Cohen, A. J.; Yang, W. Delocalization Errors in Density Functionals and Implications for Main-Group Thermochemistry. *J. Chem. Phys.* **2008**, *129*,

204112.
(20) Zhao, Y.; Truhlar, D. G. The M06 Suite of Density Functionals for Main Group Thermochemistry, Thermochemical Kinetics, Noncovalent Interactions, Excited States, and Transition Elements: Two New Functionals and Systematic Testing of Four M06-Class Functionals and 12 Other Function. *Theor. Chem. Acc.* **2008**, *120*, 215–241.
(21) Grimme, S. Semiempirical Hybrid Density Functional with Perturbative Second-Order Correlation. *J. Chem. Phys.* **2006**, *124*, 0–16.
(22) Grimme, S.; Mück-Lichtenfeld, C.; Würthwein, E. U.; Ehlers, A. W.; Goumans, T. P. M.; Lammertsma, K. Consistent Theoretical Description of 1,3-Dipolar Cycloaddition Reactions. *J. Phys. Chem. A* **2006**, *110*, 2583–2586.
(23) Piacenza, M.; Grimme, S. Systematic Quantum Chemical Study of DNA-Base Tautomers. *J. Comput. Chem.* **2004**, *25*, 83–98.
(24) Woodcock, H. L.; Schaefer, H. F.; Schreiner, P. R. Problematic Energy Differences between Cumulenes and Poly-Ynes: Does This Point to a Systematic Improvement of Density Functional Theory? *J. Phys. Chem. A* **2002**, *106*, 11923–11931.
(25) Schreiner, P. R.; Fokin, A. A.; Pascal, R. A.; Meijere, A. De. Many Density Functional Theory Approaches Fail To Give Reliable Large Hydrocarbon Isomer Energy Differences. **2006**, 10–13.
(26) Lepetit, C.; Chermette, H.; Heully, J.; Lyon, D.; Uni, V.; Umr, C. Description of Carbo - Oxocarbons and Assessment of Exchange-Correlation Functionals for the DFT Description of Carbo -Mers. **2007**, 136–149.
(27) Lee, J. S. Accurate Ab Initio Binding Energies of Alkaline Earth Metal Clusters. *J. Phys. Chem. A* **2005**, *109*, 11927–11932.
(28) Karton, A.; Martin, J. M. L. Explicitly Correlated Benchmark Calculations on C 8 H 8 Isomer Energy Separations : How Accurate Are DFT , Double-Hybrid , and Composite Ab Initio Procedures ? **2012**, *8976*.
(29) Zhao, Y.; Tishchenko, O.; Gour, J. R.; Li, W.; Lutz, J. J.; Piecuch, P.; Truhlar, D. G. Thermochemical Kinetics for Multireference Systems: Addition Reactions of Ozone. *J. Phys. Chem. A* **2009**, *113*, 5786–5799.
(30) Manna, D.; Martin, J. M. L. What Are the Ground State Structures of C 20 and C 24 ? An Explicitly Correlated Ab Initio Approach. **2016**.
(31) Friedrich, J.; Hänchen, J. Incremental CCSD(T)(F12*)|MP2: A Black Box Method to Obtain Highly Accurate Reaction Energies. *J. Chem. Theory Comput.* **2013**, *9*, 5381–5394.
(32) Friedrich, J. Efficient Calculation of Accurate Reaction Energies—Assessment of Different Models in Electronic Structure Theory. *J. Chem. Theory Comput.* **2015**, *11*, 3596–3609.
(33) You, A.; Be, M. A. Y.; In, I. Gaussian-2 Theory for Molecular Energies of First- and Second-Row Compounds Compounds. **1998**, *7221*.
(34) Curtiss, L. A.; Redfern, P. C. Assessment of Gaussian-2 and Density Functional Theories for the Computation of Enthalpies of Formation for the Computation of Enthalpies of Formation. **2000**, *1063*.
(35) Kozuch, S.; Martin, J. M. L. Halogen Bonds: Benchmarks and Theoretical Analysis. *J. Chem. Theory Comput.* **2013**, *9*, 1918–1931.
(36) Řezáč, J.; Riley, K. E.; Hobza, P. Benchmark Calculations of Noncovalent Interactions of Halogenated Molecules. *J. Chem. Theory Comput.* **2012**, *8*, 4285–4292.

(37) Schwabe, T.; Grimme, S. Double-Hybrid Density Functionals with Long-Range Dispersion Corrections: Higher Accuracy and Extended Applicability. *Phys. Chem. Chem. Phys.* **2007**, *9*, 3397–3406.
(38) Grimme, S. Seemingly Simple Stereoelectronic Effects in Alkane Isomers and the Implications for Kohn–Sham Density Functional Theory. *Angew. Chemie Int. Ed.* **2006**, *45*, 4460–4464.
(39) Goerigk, L.; Grimme, S. Efficient and Accurate Double-Hybrid-Meta-GGA Density Functionals—Evaluation with the Extended GMTKN30 Database for General Main Group Thermochemistry, Kinetics, and Noncovalent Interactions. *J. Chem. Theory Comput.* **2011**, *7*, 291–309.
(40) Grimme, S.; Steinmetz, M.; Korth, M. How to Compute Isomerization Energies of Organic Molecules with Quantum Chemical Methods. *J. Org. Chem.* **2007**, *72*, 2118–2126.
(41) Goerigk, L.; Sharma, R. The INV24 Test Set: How Well Do Quantum-Chemical Methods Describe Inversion and Racemization Barriers? *Can. J. Chem.* **2016**, *94*, 1133–1143.
(42) Huenerbein, R.; Schirmer, B.; Moellmann, J.; Grimme, S. Effects of London Dispersion on the Isomerization Reactions of Large Organic Molecules: A Density Functional Benchmark Study. *Phys. Chem. Chem. Phys.* **2010**, *12*, 6940–6948.
(43) Fogueri, U. R.; Kozuch, S.; Karton, A.; Martin, J. M. L. The Melatonin Conformer Space: Benchmark and Assessment of Wave Function and DFT Methods for a Paradigmatic Biological and Pharmacological Molecule. *J. Phys. Chem. A* **2013**, *117*, 2269–2277.
(44) Grimme, S.; Kruse, H.; Goerigk, L.; Erker, G. The Mechanism of Dihydrogen Activation by Frustrated Lewis Pairs Revisited. *Angew. Chemie Int. Ed.* **2010**, *49*, 1402–1405.
(45) Setiawan, D.; Kraka, E.; Cremer, D. Strength of the Pnicogen Bond in Complexes Involving Group Va Elements N, P, and As. **2015**.
(46) Karton, A.; O'Reilly, R. J.; Chan, B.; Radom, L. Determination of Barrier Heights for Proton Exchange in Small Water, Ammonia, and Hydrogen Fluoride Clusters with G4(MP2)-Type, MPn, and SCS-MPn Procedures-a Caveat. *J. Chem. Theory Comput.* **2012**, *8*, 3128–3136.
(47) Neese, F.; Schwabe, T.; Kossmann, S.; Schirmer, B.; Grimme, S. Assessment of Orbital-Optimized , Spin-Component Scaled Second-Order Many-Body Perturbation Theory for Thermochemistry and Kinetics. **2009**, 3060–3073.
(48) Jurečka, P.; Šponer, J.; Černý, J.; Hobza, P. Benchmark Database of Accurate (MP2 and CCSD(T) Complete Basis Set Limit) Interaction Energies of Small Model Complexes, DNA Base Pairs, and Amino Acid Pairs. *Phys. Chem. Chem. Phys.* **2006**, *8*, 1985–1993.
(49) Řezáč, J.; Riley, K. E.; Hobza, P. S66: A Well-Balanced Database of Benchmark Interaction Energies Relevant to Biomolecular Structures. *J. Chem. Theory Comput.* **2011**, *7*, 2427–2438.
(50) French, A. D.; Johnson, G. P.; Stortz, C. A. Evaluation of Density Functionals and Basis Sets for Ga. **2009**, 679–692.
(51) Karton, A.; Rabinovich, E.; Martin, J. M. L.; Ruscic, B. W4 Theory for Computational Thermochemistry: In Pursuit of Confident Sub-KJ/Mol Predictions. *J. Chem. Phys.* **2006**, *125*, 144108.
(52) Kruse, H.; Mladek, A.; Gkionis, K.; Hansen, A.; Grimme, S.; Sponer, J. Quantum Chemical Benchmark Study on 46 RNA Backbone Families Using a Dinucleotide Unit. *J. Chem. Theory Comput.* **2015**, *11*, 4972–4991.

(53) Karton, A.; Daon, S.; Martin, J. M. L. W4-11: A High-Confidence Benchmark Dataset for Computational Thermochemistry Derived from First-Principles W4 Data. *Chem. Phys. Lett.* **2011**, *510*, 165–178.
(54) Iii, W. A. G.; Park, U. V; Pennsyl, V. Evaluation of B3LYP , X3LYP , and M06-Class Density Functionals for Predicting the Binding Energies of Neutral , Protonated , and Deprotonated Water Clusters. **2009**, 1016–1026.
(55) Karton, A.; O'Reilly, R. J.; Radom, L. Assessment of Theoretical Procedures for Calculating Barrier Heights for a Diverse Set of Water-Catalyzed Proton-Transfer Reactions. *J. Phys. Chem. A* **2012**, *116*, 4211–4221.
(56) Zhao, Y.; Ng, H. T.; Peverati, R.; Truhlar, D. G. Benchmark Database for Ylidic Bond Dissociation Energies and Its Use for Assessments of Electronic Structure Methods. *J. Chem. Theory Comput.* **2012**, *8*, 2824–2834.
(57) Weigend, F.; Ahlrichs, R. Balanced Basis Sets of Split Valence, Triple Zeta Valence and Quadruple Zeta Valence Quality for H to Rn: Design and Assessment of Accuracy. *Phys. Chem. Chem. Phys.* **2005**, *7*, 3297–3305.
(58) Santra, G.; Sylvetsky, N.; Martin, J. M. L. Minimally Empirical Double-Hybrid Functionals Trained against the GMTKN55 Database: RevDSD-PBEP86-D4, RevDOD-PBE-D4, and DOD-SCAN-D4. *J. Phys. Chem. A* **2019**, *123*, 5129–5143.
(59) Sancho-García, J. C.; Brémond; Savarese, M.; Pérez-Jiménez, A. J.; Adamo, C. Partnering Dispersion Corrections with Modern Parameter-Free Double-Hybrid Density Functionals. *Phys. Chem. Chem. Phys.* **2017**, *19*, 13481–13487.
(60) Hui, K.; Chai, J.-D. SCAN-Based Hybrid and Double-Hybrid Density Functionals from Models without Fitted Parameters. *J. Chem. Phys.* **2016**, *144*, 044114.
(61) Yanai, T.; Tew, D. P.; Handy, N. C. A New Hybrid Exchange–Correlation Functional Using the Coulomb-Attenuating Method (CAM-B3LYP). *Chem. Phys. Lett.* **2004**, *393*, 51–57.
(62) Verma, P.; Wang, Y.; Ghosh, S.; He, X.; Truhlar, D. G. Revised M11 Exchange-Correlation Functional for Electronic Excitation Energies and Ground-State Properties. *J. Phys. Chem. A* **2019**, *123*, 2966–2990.
(63) Santra, G.; Martin, J. M. L. Some Observations on the Performance of the Most Recent Exchange-Correlation Functionals for the Large and Chemically Diverse GMTKN55 Benchmark. In *AIP Conference Proceedings*; 2019; p 030004.
(64) Adamo, C.; Barone, V. Toward Reliable Density Functional Methods without Adjustable Parameters: The PBE0 Model. *J. Chem. Phys.* **1999**, *110*, 6158–6170.
(65) Zhao, Y.; Truhlar, D. G. Design of Density Functionals That Are Broadly Accurate for Thermochemistry, Thermochemical Kinetics, and Nonbonded Interactions. *J. Phys. Chem. A* **2005**, *109*, 5656–5667.
(66) Becke, A. D. A New Mixing of Hartree-Fock and Local Density-Functional Theories. *J. Chem. Phys.* **1993**, *98*, 1372–1377.
(67) Mardirossian, N.; Head-Gordon, M. Mapping the Genome of Meta-Generalized Gradient Approximation Density Functionals: The Search for B97M-V. *J. Chem. Phys.* **2015**, *142*, 074111.
(68) Sun, J.; Ruzsinszky, A.; Perdew, J. Strongly Constrained and Appropriately Normed Semilocal Density Functional. *Phys. Rev. Lett.* **2015**, *115*, 036402.
(69) Perdew, J. P.; Ruzsinszky, A.; Csonka, G. I.; Constantin, L. A.; Sun, J. Workhorse Semilocal

Density Functional for Condensed Matter Physics and Quantum Chemistry. *Phys. Rev. Lett.* **2009**, *103*, 026403.
(70) Perdew, J. P.; Burke, K.; Ernzerhof, M. Generalized Gradient Approximation Made Simple. *Phys. Rev. Lett.* **1996**, *77*, 3865–3868.
(71) Perdew, J. P.; Ernzerhof, M.; Burke, K. [ERRATA] Generalized Gradient Approximation Made Simple. *Phys. Rev. Lett.* **1996**, *77*, 3865–3868.
(72) Slater, J. C. A Simplification of the Hartree-Fock Method. *Phys. Rev.* **1951**, *81*, 385–390.
(73) Perdew, J. P.; Wang, Y. Accurate and Simple Analytic Representation of the Electron-Gas Correlation Energy. *Phys. Rev. B* **1992**, *45*, 13244–13249.
(74) Martin, J. M. L.; Santra, G. Empirical Double-Hybrid Density Functional Theory: A 'Third Way' in Between WFT and DFT. *Isr. J. Chem.* **2020**, *60*, 787–804.
(75) Axilrod, B. M.; Teller, E. Interaction of the van Der Waals Type Between Three Atoms. *J. Chem. Phys.* **1943**, *11*, 299–300.
(76) Muto, Y. Force between Nonpolar Molecules. *Proc. Physico-Mathematical Soc. Japan* **1943**, *17*, 629–631.
