# Supplementary material for: Correction to “Exploring Avenues beyond Revised DSD Functionals: I. Range Separation, with xDSD as a Special Case”
Source: J Phys Chem A. 2024 Jan 30;128(5):974–5. doi: 10.1021/acs.jpca.4c00309 (PMC11027754; doi:10.1021/acs.jpca.4c00309)
Supplement: Supplementary file 1 — jp4c00309_si_001.pdf [file jp4c00309_si_001.pdf]

Electronic Supporting Information (ESI)

for

Correction to “Exploring Avenues Beyond Revised DSD Functionals: I. range separation, with xDSD as a special case” [*J. Phys. Chem. A* 2021, *125* (21), 4614-4627. DOI: 10.1021/acs.jpca.1c01294.]

Golokesh Santra,[†, #] Minsik Cho,[†, §] and Jan M.L. Martin[,†]*

[†]Department of Organic Chemistry, Weizmann Institute of Science, 7610001 Reḥovot, Israel.
Email: gershom@weizmann.ac.il

[#] Max-Planck-Institut für Kohlenforschung, Kaiser-Wilhelm-Platz 1, 45470 Mülheim an der Ruhr, Germany.

[§] Department of Chemistry, Massachusetts Institute of Technology, Cambridge, Massachusetts 02139, USA.

SI.1. Division of WTMAD2 into five major subcategories:

Table S1: Division of total WTMAD2(kcal/mol) for xDSD- and xDOD-D4 functionals into five major subsets: Small Molecule Thermochemistry (THERMO), Intermolecular Interactions (INTERMOL), Conformers/Intramolecular Interactions (CONF), Barrier Heights (BARRIERS), Small Molecule Thermochemistry (THERMO) and Large-species Reaction Energies (LARGE)

Functionals	THERMO	BARRIERS	LARGE	CONF	INTERMOL	WTMAD2
xDSD$_{75}$-PBEP86-D4	0.508	0.251	0.493	0.409	0.457	2.119
xDSD$_{69}$-PBEP86-D4	0.570	0.247	0.389	0.703	0.491	2.400
xDSD$_{74}$-PBEB95-D4	0.494	0.291	0.479	0.453	0.530	2.247
xDSD$_{77}$-BLYP-D4	0.570	0.314	0.541	0.421	0.533	2.379
xDSD$_{69}$-SCAN-D4	0.580	0.270	0.472	0.404	0.477	2.203
xDSD$_{72}$-PBEPW91-D4	0.598	0.272	0.473	0.412	0.484	2.238
xDSD$_{72}$-PBEPBE-D4	0.571	0.234	0.512	0.408	0.470	2.196
xDOD$_{72}$-PBEP86-D4	0.608	0.215	0.378	0.772	0.517	2.490
xDOD$_{69}$-PBEB95-D4	0.677	0.290	0.520	0.480	0.576	2.542
xDOD$_{74}$-BLYP-D4	0.583	0.309	0.541	0.423	0.529	2.384
xDOD$_{69}$-SCAN-D4	0.604	0.244	0.473	0.414	0.484	2.219
xDOD$_{69}$-PBEPW91-D4	0.622	0.250	0.466	0.418	0.488	2.243
xDOD$_{69}$-PBEPBE-D4	0.508	0.251	0.493	0.409	0.457	2.119

SI.2. MAD, MSD, RMSD and division of WTMAD2 into 55 subsets

Table S2: MAD, MSD and RMSD as well as breakdown of total WTMAD2 by each subset for $xDSD_{75}$-PBEP86-D4

subs.name	MAD	MSD	RSMD	dWTMAD2	5MAD/4RMSD
ACONF	0.052	0.052	0.068	0.0163	0.9652
ADIM6	0.356	-0.356	0.393	0.0241	1.1313
AHB21	0.238	-0.219	0.341	0.0084	0.8722
AL2X6	1.407	-1.407	1.421	0.0089	1.2375
ALK8	1.825	-1.398	2.307	0.0088	0.9887
ALKBDE10	2.79	-2.258	3.465	0.0105	1.0066
AMINO20X4	0.117	-0.042	0.15	0.146	0.9789
BH76RC	0.72	0.178	0.936	0.0383	0.9612
BH76	0.951	0.68	1.977	0.1472	0.6012
BHDIV10	0.656	-0.091	0.79	0.0055	1.0377
BHPERI	0.708	-0.599	0.815	0.0334	1.0859
BHROT27	0.087	0.083	0.118	0.0142	0.9181
BSR36	0.776	-0.776	0.8	0.0654	1.2128
BUT14DIOL	0.058	0.058	0.068	0.0505	1.0721
C60ISO	7.44	-7.407	9.837	0.0258	0.9454
CARBHB12	0.304	0.304	0.385	0.0229	0.9872
CDIE20	0.328	0.328	0.386	0.0613	1.0604
CHB6	0.717	-0.68	0.892	0.0061	1.005
DARC	0.37	0.11	0.482	0.006	0.9585
DC13	1.841	0.957	2.464	0.0165	0.934
DIPCS10	4.753	-4.753	5.007	0.0028	1.1865
FH51	0.647	-0.033	0.877	0.0404	0.9219
G21EA	2.699	-2.529	3.118	0.0761	1.0821
G21IP	2.116	-1.316	2.535	0.0112	1.0434
G2RC	1.338	0.35	1.811	0.0248	0.9237
HAL59	0.228	0.142	0.316	0.1112	0.903
HEAVY28	0.092	0.013	0.136	0.0789	0.8475
HEAVYSB11	1.105	-1.105	1.322	0.0079	1.0448
ICONF	0.114	-0.026	0.149	0.0224	0.9516
IDISP	0.66	0.237	0.785	0.0106	1.0504
IL16	0.235	0.235	0.289	0.0013	1.0151
INV24	0.697	0.068	1.175	0.0199	0.7419
ISO34	0.382	-0.129	0.515	0.0338	0.9284
ISOL24	1.02	-0.169	1.456	0.0423	0.8755
MB16-43	10.515	-10.234	11.776	0.0366	1.1161
MCONF	0.094	0.053	0.115	0.0365	1.0207
NBPRC	0.328	-0.031	0.474	0.003	0.866
PA26	1.298	1.298	1.63	0.0068	0.9953
PAREL	0.357	0.065	0.583	0.0585	0.7657
PCONF21	0.111	-0.01	0.128	0.0467	1.0829
PNICO23	0.078	0.03	0.091	0.0158	1.0622
PX13	1.158	-1.158	1.27	0.0171	1.1398
RC21	1.764	-1.52	2.227	0.0393	0.9903
RG18	0.076	-0.041	0.098	0.0893	0.9718
RSE43	0.76	0.746	1.41	0.163	0.6738
S22	0.112	0.045	0.141	0.0128	0.9992
S66	0.17	-0.009	0.207	0.0779	1.0261
SCONF	0.061	-0.003	0.095	0.0085	0.8012
SIE4X4	3.844	3.844	4.443	0.0692	1.0815
TAUT15	0.516	-0.264	0.573	0.0963	1.1258
UPU23	0.467	0.351	0.578	0.0712	1.0096
W4-11	2.07	-0.647	2.977	0.0358	0.8691
WATER27	0.687	0.641	0.872	0.0087	0.9851
WCPT18	0.721	-0.545	0.904	0.0141	0.9961
YBDE18	0.843	0.811	1.357	0.0117	0.7771
**GMTKN55**				**2.1187**	

Table S3: MAD, MSD and RMSD as well as breakdown of total WTMAD2 by each subset for xDOD$_{72}$-PBEP86-D4

subs.name	MAD	MSD	RSMD	dWTMAD2	5MAD/4RMSD
ACONF	0.036	0.031	0.046	0.0111	0.9671
ADIM6	0.307	-0.307	0.343	0.0208	1.1174
AHB21	0.204	-0.175	0.295	0.0072	0.8629
AL2X6	1.266	-1.266	1.284	0.008	1.2333
ALK8	1.703	-1.283	2.247	0.0083	0.9474
ALKBDE10	2.875	-2.708	3.553	0.0108	1.0114
AMINO20X4	0.123	-0.051	0.159	0.1524	0.9645
BH76RC	0.928	0.138	1.066	0.0494	1.0887
BH76	0.984	0.479	1.823	0.1524	0.6751
BHDIV10	0.531	0.029	0.641	0.0044	1.0346
BHPERI	0.379	0.157	0.487	0.0179	0.971
BHROT27	0.073	0.058	0.094	0.0119	0.9723
BSR36	1.241	-1.241	1.324	0.1046	1.1716
BUT14DIOL	0.058	0.057	0.068	0.05	1.0582
C60ISO	6.073	-6.011	8.096	0.0211	0.9376
CARBHB12	0.285	0.285	0.357	0.0215	0.9974
CDIE20	0.282	0.26	0.351	0.0528	1.0041
CHB6	0.772	-0.703	0.931	0.0066	1.0362
DARC	0.317	0.158	0.389	0.0052	1.0192
DC13	1.902	0.458	2.343	0.0171	1.0148
DIPCS10	4.101	-4.101	4.408	0.0024	1.1629
FH51	0.743	0.082	0.947	0.0463	0.9811
G21EA	2.635	-2.319	3.072	0.0743	1.0723
G21IP	2.012	-1.035	2.482	0.0107	1.0134
G2RC	1.533	0.49	2.071	0.0283	0.9252
HAL59	0.244	0.166	0.326	0.1188	0.936
HEAVY28	0.11	0.04	0.161	0.0944	0.8562
HEAVYSB11	1.087	-1.084	1.362	0.0078	0.998
ICONF	0.118	-0.055	0.156	0.0232	0.9436
IDISP	0.626	0.303	0.737	0.01	1.0607
IL16	0.254	0.25	0.302	0.0014	1.0522
INV24	0.7	0.125	1.229	0.02	0.7119
ISO34	0.38	-0.23	0.544	0.0336	0.8728
ISOL24	1.004	-0.316	1.439	0.0417	0.8723
MB16-43	13.708	-13.658	15.512	0.0477	1.1046
MCONF	0.075	0.022	0.091	0.029	1.0292
NBPRC	0.35	-0.11	0.557	0.0032	0.7856
PA26	1.688	1.688	1.978	0.0088	1.0671
PAREL	0.333	0.031	0.575	0.0546	0.7249
PCONF21	0.109	-0.013	0.132	0.0458	1.0305
PNICO23	0.095	0.071	0.12	0.0194	0.9872
PX13	0.996	-0.987	1.117	0.0147	1.1146
RC21	1.471	-1.248	1.831	0.0328	1.0041
RG18	0.072	-0.033	0.091	0.085	0.9918
RSE43	0.703	0.676	1.245	0.1509	0.7065
S22	0.108	0.041	0.137	0.0124	0.9874
S66	0.163	0.004	0.2	0.0747	1.0224
SCONF	0.078	-0.057	0.113	0.011	0.865
SIE4X4	4.409	4.409	5.189	0.0793	1.0622
TAUT15	0.664	-0.407	0.743	0.1239	1.1162
UPU23	0.495	0.385	0.605	0.0754	1.0232
W4-11	2.836	0.067	3.708	0.049	0.9559
WATER27	0.649	0.612	0.841	0.0082	0.9643
WCPT18	0.653	-0.247	0.772	0.0127	1.0572
YBDE18	0.775	0.653	1.175	0.0107	0.8243
**GMTKN55**				**2.1957**	

SI.4. MAD and RMSD of POLYPYR21

Table S4: Mean Absolute Deviations (in kcal/mol) and Root Mean Squared Deviations (in kcal/mol) for the new xDSD(DOD) and ωDSD(DOD) functionals evaluated against POLYPYR21. Only the MADs and RMSDs of xDSD- and xDOD-D4 are updated. Everything else stays same as in ref.[1]

Functionals	MAD (kcal/mol)	RMSD (kcal/mol)		
		Total	Möbius Structures	Hückel & figure-eight Structures
$xDSD_{72}$-PBEPBE-D3BJ	2.31	3.33	5.36	0.90
$xDOD_{69}$-PBEPBE-D3BJ	1.69	2.37	3.68	0.76
$xDSD_{74}$-PBEB95-D3BJ	2.50	3.59	5.73	0.95
$xDOD_{69}$-PBEB95-D3BJ	1.30	1.75	2.34	0.81
$xDSD_{72}$-PBEPW91-D3BJ	2.38	3.44	5.53	0.91
$xDOD_{69}$-PBEPW91-D3BJ	1.63	2.27	3.47	0.75
$xDSD_{69}$-SCAN-D3BJ	1.96	2.73	4.29	0.86
$xDOD_{69}$-SCAN-D3BJ	1.63	2.24	3.42	0.80
$xDSD_{77}$-BLYP-D3BJ	3.08	4.51	7.28	1.14
$xDOD_{74}$-BLYP-D3BJ	1.19	1.64	2.13	0.75
$xDSD_{75}$-PBEP86-D3BJ	2.46	3.58	5.74	0.90
$xDOD_{72}$-PBEP86-D3BJ	1.42	1.96	2.88	0.70
$xDSD_{72}$-PBEPBE-D4	2.10	2.98	4.59	0.83
$xDOD_{69}$-PBEPBE-D4	1.71	2.39	3.53	0.80
$xDSD_{74}$-PBEB95-D4	2.20	3.14	4.61	0.94
$xDOD_{69}$-PBEB95-D4	1.54	2.13	2.55	1.05
$xDSD_{72}$-PBEPW91-D4	2.20	3.13	4.83	0.85
$xDOD_{69}$-PBEPW91-D4	1.68	2.35	3.43	0.80
$xDSD_{69}$-SCAN-D4	1.91	2.69	4.01	0.86
$xDOD_{69}$-SCAN-D4	1.64	2.29	3.28	0.83
$xDSD_{77}$-BLYP-D4	3.03	4.43	6.95	1.01
$xDOD_{74}$-BLYP-D4	1.31	1.87	2.24	0.86
$xDSD_{75}$-PBEP86-D4	2.41	3.47	5.41	0.84
$xDOD_{72}$-PBEP86-D4	1.46	2.06	2.84	0.76
ωDSD_{72}-PBEP86-D3BJ(ω=0.13)	1.61	2.34	3.77	0.72
ωDOD_{72}-PBEP86-D3BJ(ω=0.08)	1.28	1.85	3.09	0.63
ωDSD_{69}-PBEP86-D3BJ(ω=0.16)	0.92	1.34	2.12	0.54
ωDOD_{69}-PBEP86-D3BJ(ω=0.10)	0.70	1.00	1.56	0.49
ωDSD_{66}-PBEP86-D3BJ(ω=0.18)	0.53	0.72	1.09	0.44
ωDOD_{66}-PBEP86-D3BJ(ω=0.15)	0.38	0.49	0.69	0.38
ωDSD_{63}-PBEP86-D3BJ(ω=0.20)	0.97	1.33	1.86	0.60
ωDOD_{63}-PBEP86-D3BJ(ω=0.16)	0.78	1.06	1.41	0.55
ωDSD_{60}-PBEP86-D3BJ(ω=0.22)	0.38	0.49	0.45	0.44
ωDOD_{60}-PBEP86-D3BJ(ω=0.18)	**0.35**	**0.45**	0.49	0.41
ωDSD_{57}-PBEP86-D3BJ(ω=0.22)	0.44	0.60	0.95	0.38
ωDOD_{57}-PBEP86-D3BJ(ω=0.20)	0.55	0.75	1.26	0.36
ωDSD_{72}-PBEP86-D4(ω=0.13)	1.49	2.16	3.32	0.59
ωDOD_{72}-PBEP86-D4(ω=0.08)	1.08	1.58	2.36	0.50
ωDSD_{69}-PBEP86-D4(ω=0.16)	0.84	1.25	1.80	0.46
ωDOD_{69}-PBEP86-D4(ω=0.10)	0.56	0.84	1.08	0.43
ωDSD_{66}-PBEP86-D4(ω=0.18)	0.43	0.59	0.67	0.40
ωDOD_{66}-PBEP86-D4(ω=0.15)	0.42	0.55	0.64	0.42
ωDSD_{63}-PBEP86-D4(ω=0.20)	0.99	1.39	1.70	0.64
ωDOD_{63}-PBEP86-D4(ω=0.16)	0.82	1.16	1.34	0.59
ωDSD_{60}-PBEP86-D4(ω=0.22)	0.45	0.65	0.41	0.50
ωDOD_{60}-PBEP86-D4(ω=0.18)	0.43	0.59	0.47	0.48
ωDSD_{57}-PBEP86-D4(ω=0.22)	0.55	0.55	0.99	0.48
ωDOD_{57}-PBEP86-D4(ω=0.20)	0.63	0.83	1.32	0.48
ωB97M(2)	0.48	0.63	0.82	0.55
ωB2PLYP	0.97	1.28	2.10	0.62
ωB2GP-PLYP	0.61	0.78	0.99	0.57

SI.5. MAD and RMSD of MPCONF196

Table S5: Mean Absolute Deviations (in kcal/mol) and Root Mean Squared Deviations (in kcal/mol) of conformational energies for various DHDFs evaluated against MPCONF196 data set. Only the MADs and RMSDs of xDSD- and xDOD-D4 are updated. Everything else stays same as in ref.[1]

Functionals	MAD (kcal/ Mol)	RMSD(kcal/mol)			
		Total	Small[a]	Medium[b]	Large[c]
ωB97X-V	0.45	0.73	0.19	0.50	1.36
ωB97M-V	0.55	0.87	0.27	0.61	1.60
ωB97M(2)	0.37	0.62	0.16	0.38	1.17
revDSD-PBEP86-D3BJ	0.29	0.46	0.17	0.25	0.89
revDSD-PBEP86-D4	0.26	0.38	0.17	0.25	0.69
revDOD-PBEP86-D3BJ	0.29	0.47	0.17	0.26	0.90
revDOD-PBEP86-D4	0.26	0.38	0.17	0.26	0.67
revDSD-PBEPBE-D3BJ	0.34	0.53	0.22	0.43	0.90
revDSD-PBEPBE-D4	0.29	0.44	0.17	0.41	0.72
revDOD-PBEPBE-D3BJ	0.34	0.53	0.22	0.44	0.90
revDOD-PBEPBE-D4	0.29	0.44	0.17	0.41	0.72
DOD66-SCAN-D3BJ	0.32	0.49	0.21	0.29	0.92
DOD66-SCAN-D4	0.27	0.38	0.20	0.30	0.64
revDSD-PBEB95-D3BJ	0.44	0.61	0.35	0.45	1.05
revDSD-PBEB95-D4	0.36	0.50	0.29	0.43	0.80
revDOD-PBEB95-D3BJ	0.46	0.64	0.37	0.48	1.08
revDOD-PBEB95-D4	0.36	0.51	0.29	0.44	0.81
revDSD-BLYP-D3BJ	0.35	0.58	0.16	0.31	1.11
revDSD-BLYP-D4	0.29	0.44	0.18	0.29	0.81
revDOD-BLYP-D3BJ	0.36	0.60	0.16	0.34	1.15
revDOD-BLYP-D4	0.30	0.46	0.20	0.31	0.83
revDSD-PBEPW91-D3BJ	0.33	0.50	0.21	0.32	0.92
revDSD-PBEPW91-D4	0.27	0.40	0.18	0.31	0.70
revDOD-PBEPW91-D3BJ	0.32	0.50	0.21	0.32	0.91
revDOD-PBEPW91-D4	0.28	0.40	0.17	0.32	0.70
xDSD$_{75}$-PBEP86-D3BJ	0.31	0.50	0.16	0.27	0.96
xDSD$_{75}$-PBEP86-D4	0.26	0.39	0.17	0.26	0.71
xDOD$_{72}$-PBEP86-D3BJ	0.31	0.51	0.16	0.29	0.97
xDOD$_{72}$-PBEP86-D4	0.26	0.39	0.18	0.26	0.70
xDSD$_{69}$-SCAN-D3BJ	0.32	0.51	0.20	0.30	0.96
xDOD$_{69}$-SCAN-D3BJ	0.34	0.53	0.21	0.31	0.99
xDSD$_{69}$-SCAN-D4	0.27	0.39	0.20	0.30	0.68
xDOD$_{69}$-SCAN-D4	0.27	0.39	0.20	0.30	0.67
xDSD$_{77}$-BLYP-D3BJ	0.36	0.59	0.18	0.32	1.13
xDSD$_{77}$-BLYP-D4	0.29	0.43	0.19	0.28	0.78
xDOD$_{74}$-BLYP-D3BJ	0.36	0.60	0.17	0.35	1.14
xDOD$_{74}$-BLYP-D4	0.31	0.45	0.21	0.31	0.82
xDSD$_{72}$-PBEPBE-D3BJ	0.34	0.53	0.20	0.32	0.99
xDSD$_{72}$-PBEPBE-D4	0.28	0.42	0.18	0.31	0.74
xDOD$_{69}$-PBEPBE-D3BJ	0.34	0.53	0.20	0.33	0.97
xDOD$_{69}$-PBEPBE-D4	0.28	0.42	0.18	0.32	0.73
xDSD$_{74}$-PBEB95-D3BJ	0.46	0.66	0.35	0.46	1.16
xDSD$_{74}$-PBEB95-D4	0.37	0.52	0.29	0.41	0.87
xDOD$_{69}$-PBEB95-D3BJ	0.49	0.69	0.38	0.51	1.19
xDOD$_{69}$-PBEB95-D4	0.38	0.54	0.29	0.45	0.89
xDSD$_{72}$-PBEPW91-D3BJ	0.33	0.52	0.19	0.31	0.99
xDSD$_{72}$-PBEPW91-D4	0.28	0.42	0.18	0.30	0.74
xDOD$_{69}$-PBEPW91-D3BJ	0.33	0.52	0.20	0.32	0.97
xDOD$_{69}$-PBEPW91-D4	0.28	0.41	0.18	0.31	0.73
ωDSD$_{72}$-PBEP86-D3BJ(ω=0.13)	0.32	0.53	0.17	0.29	1.01
ωDSD$_{72}$-PBEP86-D4(ω=0.13)	0.27	0.40	0.18	0.27	0.73
ωDOD$_{72}$-PBEP86-D3BJ(ω=0.08)	0.32	0.53	0.17	0.30	1.00
ωDOD$_{72}$-PBEP86-D4(ω=0.08)	0.27	0.40	0.18	0.27	0.71
ωDSD$_{69}$-PBEP86-D3BJ(ω=0.16)	0.32	0.53	0.17	0.29	1.01
ωDSD$_{69}$-PBEP86-D4(ω=0.16)	0.27	0.40	0.17	0.27	0.72
ωDOD$_{69}$-PBEP86-D3BJ(ω=0.10)	0.32	0.52	0.17	0.30	1.00
ωDOD$_{69}$-PBEP86-D4(ω=0.10)	0.27	0.39	0.18	0.27	0.71
ωDSD$_{66}$-PBEP86-D3BJ(ω=0.18)	0.32	0.53	0.17	0.29	1.01
ωDSD$_{66}$-PBEP86-D4(ω=0.18)	0.27	0.41	0.17	0.27	0.74
ωDOD$_{66}$-PBEP86-D3BJ(ω=0.15)	0.33	0.53	0.17	0.31	1.01
ωDOD$_{66}$-PBEP86-D4(ω=0.15)	0.27	0.40	0.17	0.28	0.72
ωDSD$_{63}$-PBEP86-D3BJ(ω=0.20)	0.32	0.53	0.17	0.30	1.01
ωDSD$_{63}$-PBEP86-D4(ω=0.20)	0.27	0.41	0.17	0.28	0.74
ωDOD$_{63}$-PBEP86-D3BJ(ω=0.16)	0.33	0.53	0.17	0.31	1.01
ωDOD$_{63}$-PBEP86-D4(ω=0.16)	0.27	0.41	0.17	0.28	0.74
ωDSD$_{60}$-PBEP86-D3BJ(ω=0.22)	0.32	0.53	0.17	0.30	1.01
ωDSD$_{60}$-PBEP86-D4(ω=0.22)	0.27	0.40	0.17	0.27	0.74
ωDOD$_{60}$-PBEP86-D3BJ(ω=0.18)	0.32	0.53	0.17	0.31	1.00
ωDOD$_{60}$-PBEP86-D4(ω=0.18)	0.27	0.41	0.17	0.29	0.74
ωDSD$_{57}$-PBEP86-D3BJ(ω=0.22)	0.35	0.57	0.19	0.35	1.08
ωDSD$_{57}$-PBEP86-D4(ω=0.22)	0.27	0.43	0.16	0.30	0.79
ωDOD$_{57}$-PBEP86-D3BJ(ω=0.20)	0.33	0.54	0.18	0.32	1.02
ωDOD$_{57}$-PBEP86-D4(ω=0.20)	0.27	0.41	0.17	0.29	0.73
ωB2PLYP	0.55	0.78	0.40	0.71	1.25
ωB2GP-PLYP	0.49	0.74	0.27	0.65	1.25
PBE0-D3(0)[d]	0.50	0.64	0.50	0.50	0.93
PBE-D3(0)[d]	0.69	0.83	0.78	0.70	1.04

[a]Small subsets: FGG, GGF, WG, WGG, and GFA; [b]Medium subsets: POXTRD, CAMVES, COHVAW, CHPSAR, and Cpd_B; [c]Large subsets: Cpd_A, SANGLI and YIVNOG; [d]MAD and RMSD values are taken from ref.[2]

SI.6. MAD for CHAL336 and four subsets.

Table S6: Mean Absolute Deviation (in kcal/mol) statistics of $xDSD_{75}$-PBEP86-D4 and $xDOD_{72}$-PBEP86-D4 for the full CHAL336 dataset and it's four subset. [red (worst) via yellow to green (best)]

Basis set	Functional	MAD (kcal/mol)				
		CHAL336	Chalcogen-Chalcogen	Chalcogen-Nitrogen	Chalcogen-π	Chalcogen-Halogen
ma-def2-QZVPP	$xDSD_{75}$-PBEP86-D4	0.46	0.65	0.44	0.17	0.37
	$xDOD_{72}$-PBEP86-D4	0.44	0.58	0.44	0.18	0.37
ma-{T,Q}	$xDSD_{75}$-PBEP86-D4	0.35	0.34	0.36	0.20	0.40
	$xDOD_{72}$-PBEP86-D4	0.34	0.34	0.36	0.18	0.36

SI.7. Figures

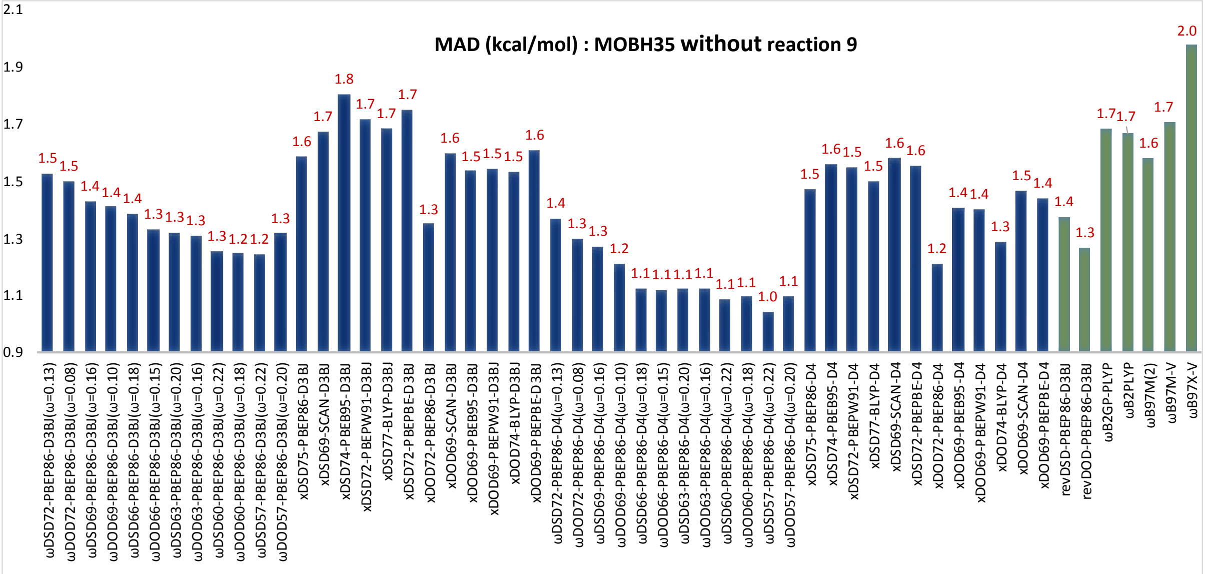

Figure S1: MAD (kcal/mol) for our new xDSD and ωDSD functionals tested against modified MOBH35. Only the MADs of xDSD- and xDOD-D4 are updated. Everything else stays same as in ref.[1]

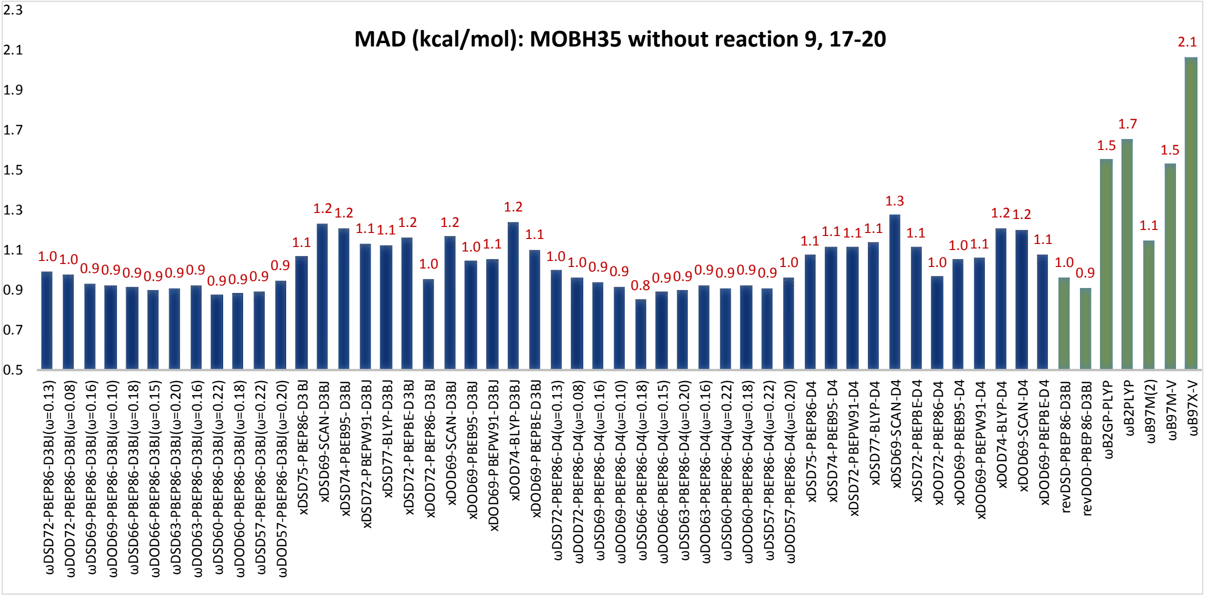

Figure S2: MAD (kcal/mol) statistics for xDSD and ωDSD functionals evaluated against modified MOBH35. Only the MADs of xDSD- and xDOD-D4 are updated. Everything else stays same as in ref.[1]

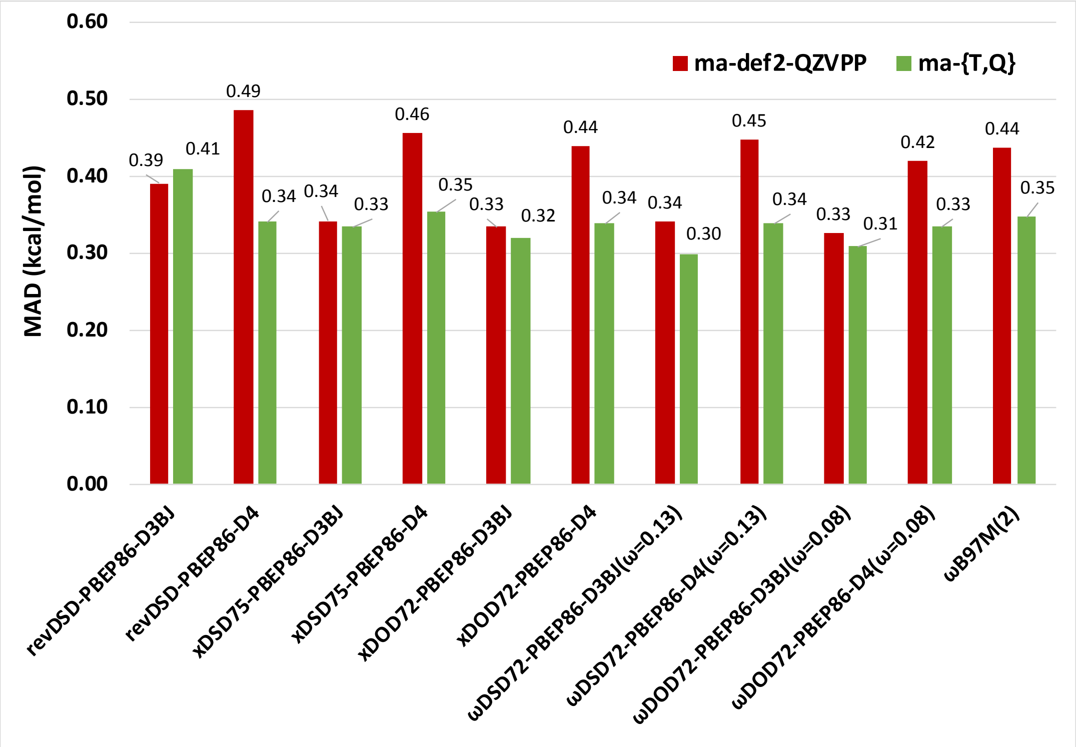

Figure S3: MAD (kcal/mol) statistics of the selected global and range separated DSD(DOD)-functionals for the CHAL336 benchmark set. Only the MADs of xDSD- and xDOD-D4 are updated. Everything else stays same as in ref.[1]

References:

(1) Santra, G.; Cho, M.; Martin, J. M. L. Exploring Avenues beyond Revised DSD Functionals: I. Range Separation, with xDSD as a Special Case. *J. Phys. Chem. A* **2021**, *125* (21), 4614-4627. DOI: 10.1021/acs.jpca.1c01294.
(2) Řezáč, J.; Bím, D.; Gutten, O.; Rulíšek, L. Toward Accurate Conformational Energies of Smaller Peptides and Medium-Sized Macrocycles: MPCONF196 Benchmark Energy Data Set. *J. Chem. Theory Comput.* **2018**, *14*, 1254. DOI: 10.1021/acs.jctc.7b01074.
